# Epidemicity conditions in spatial models of infectious diseases

**DOI:** 10.1007/s40574-025-00497-0

**Published:** 2025-07-29

**Authors:** Marino Gatto, Cristiano Trevisin, Lorenzo Mari, Damiano Pasetto, Enrico Bertuzzo, Renato Casagrandi, Andrea Rinaldo

**Affiliations:** 1 Dipartimento di Elettronica, Informazione e Bioingegneria, Politecnico di Milano, Via G. Ponzio 34/5, Milan, 20133 Italy; 2 Laboratory of Ecohydrology, École Polytechnique Fédérale de Lausanne, Station 2, Lausanne, 1015 Switzerland; 3 Sorbonne Université, INSERM, Pierre Louis Institute of Epidemiology and Public Health, 27 Rue Chaligny, Paris, 75012 France; 4 Department of Environmental Sciences, Informatics and Statistics, Ca’ Foscari University of Venice, Via Torino 155, Venezia Mestre, 30170 Italy; 5 Department of Environmental Sciences, Informatics and Statistics, Università di Padova, Via Marzolo 9, Padova, 35131 Italy

**Keywords:** Epidemiology, Space-explicit models, Reactivity, Reproduction number

## Abstract

We review the work that has been conducted in the past years to understand when and why significant epidemics may arise even though a disease is not bound to become endemic in the long run i.e. subthreshold outbreaks. In particular, we describe the methods that allow the analysis of such outbreaks not only in time but also in space. To that end, we introduce a broad class of spatially explicit mathematical models of water- and air-borne diseases. These models consist of coupled ordinary differential or difference equations. The spatial coupling is obtained via suitable networks that describe connectivity via waterways or human mobility, or both (multiplex networks). We derive exact epidemicity conditions under which the disease-free equilibrium, although asymptotically stable, may be characterized by bursts of infections possibly coalescing, epitomized by transient increases of the infected compartments given suitable perturbations. The growth of infections is quantified by means of $\ell ^1$- or $\ell ^2$-norms. We show that epidemicity is guaranteed whenever the dominant eigenvalue of a suitable Hermitian matrix is positive (continuous-time models) or larger than unity (in discrete-time models), while the corresponding reproduction number (quantified as the spectral radius of the next-generation matrix) is smaller than unit. We also apply the methodology to two relevant case studies: the 2010 cholera epidemic in Haiti and the 2020 COVID-19 outbreak in Italy.

## Introduction

In 1997, the seminal work by Neubert and Caswell [1] introduced the concept of reactivity to ecology. The idea is that, while the long-term dynamics of an ecological community is determined by the stability of its equilibria (or, more in general, attractors), finding conditions for investigating the nature of short-term transient dynamics immediately after a perturbation can be, in some cases, at least as important. For example, some alien species may be characterized by boom-and-bust demography [2, 3], in which an initial large increase in population abundance after invasion is followed by a collapse. In such cases, the damage inflicted by alien organisms can be quite large even if the stable equilibrium corresponds to the extinction of the invasive species.

Similarly, in disease ecology, conditions that guarantee that a disease cannot become endemic do not in general prevent the possible outbreak of a large transient epidemic, which might claim a large number of lives. Usually, the condition most frequently cited to epidemiologically classify a disease, be it more or less dangerous, is that the basic or the effective reproduction number (RN) be smaller than unity, a condition which confers the so-called herd immunity. However, herd immunity is not sufficient to prevent subthreshold bursts of epidemic activity, that may occur even if RN $< 1$ [4, 5], nor avoid stuttering transmission chains [6, 7] or wavelets [8]. Sometimes, peak disease prevalence may be orders of magnitude larger than the one eventually observed after the pathogen has possibly become endemic. The importance of preventing pathogens from even temporarily spreading within a population is testified, for example, by MERS-CoV outbreaks in the Middle East [9] or measles in the US that occur despite control efforts [10]. Therefore, it might be useful to introduce another epidemiological indicator, that we have termed epidemicity [11], which is linked to the concept of generalized reactivity [12].

Here we review our work on outbreaks of subthreshold epidemics in a space-explicit context with reference mainly to water-related and air-borne pathogens, and with specific examples drawn from cholera and COVID-19 epidemics. In terms of the epidemiological models employed, we will explore two possible approaches: the former is based on compartmental models, the latter on integro-differential equations, possibly discretized in time to match the available data.

## General background

In this section, we introduce the fundamental concepts that are the basis of our approach: the definition and basics of epidemicity analysis and the topology of the spatial networks.

### Epidemicity analysis

#### Ecological reactivity

Neubert and Caswell [1] proposed a useful method to characterize the transient dynamics associated with a stable equilibrium of a linear(ized) system of ODEs. Given an asymptotically stable linear system $d\textbf{x} / dt = \textbf{A} \textbf{x}$, where $\textbf{A}$ is the $n \times n$ state matrix (or the Jacobian in the nonlinear case) and $\textbf{x}$ is the difference between the system state and the equilibrium (with $\textbf{x}(t) = \mathbf {x_0}$ being the non-zero initial perturbation at $t = 0$), they defined reactivity as the maximum initial amplification rate of small perturbations to the equilibrium, evaluated over all possible local perturbations. The initial magnitude and the eventual amplification of the perturbations were quantified through the Euclidean norm of the full state vector. Neubert and Caswell [1] also showed that reactivity corresponds to the dominant eigenvalue of the Hermitian part of matrix $\textbf{A}$, i.e., $H(\textbf{A}) = (\textbf{A} + \textbf{A}^T) / 2$, with *T* indicating matrix transposition.

#### Generalized reactivity

The original definition of reactivity thus relies on an intrinsically isotropic approach which values all components of $\textbf{x}$ equally. This may be a drawback in epidemiological applications, where one is interested in the reactivity of those components of the system state pertaining to infection (such as infected and/or infectious hosts, environmental pathogen concentrations, and parasite loads), not to other state variables (e.g., susceptible hosts). Mari et al. [12] proposed an extension of the basic definition of reactivity (general reactivity, or g-reactivity) based on the dynamic analysis of a suitably defined system output obtained as a linear transformation of the state. In epidemiological applications, the system output may correspond to one or more linear combinations of the variables pertaining to the infection-related components of the system state. For instance, in a spatial SIR (numbers of susceptible-infected-recovered in different sites) model, one might choose as output the total number of infected people, or the vector whose components are the number of infected people in each site. The choice is subjective and linked to the goal pursued via the epidemiological analysis, such as forecasting the future disease incidence or planning containment measures.

#### Conditions for g-reactivity and epidemicity

A stable equilibrium (this might be, in epidemiological applications, the *disease-free equilibrium*, DFE, when infection reproduction is subcritical) is more precisely classified as g-reactive if there exist small perturbations that can lead to a transient growth in the Euclidean norm of a suitable system output vector $\textbf{y}$ that is linearly linked to the full state of the system. In formulas, this reads as $\textbf{y} = \textbf{C} \textbf{x}$, where $\textbf{C}$ is a full-rank $q \times n$ real matrix ($q \le n$). To capture transient epidemic behavior, the output transformation should include all and only the infection-related variables of the system. As we said above the choice of the matrix $\textbf{C}$, hence of the linear combination, is subjective. However, the matrix must be full rank, because otherwise one or more output variables would just be linear combinations of other output variables. In any case, it should be clear that the g-reactivity properties of the DFE do depend on the choice of $\textbf{C}$. The DFE might switch from g-reactive to non-g-reactive if one chooses a different $\textbf{C}$. Mari et al. [12] showed that a stable steady state is g-reactive if

1 $$\begin{aligned} \lambda _{\max } \left( H(\textbf{C}^T \textbf{C} \textbf{A}) \right) > 0 , \end{aligned}$$

i.e., if the dominant eigenvalue $\lambda _{\max }$ of matrix $H(\textbf{C}^T \textbf{C} \textbf{A})$ is positive.

In fact, noting that $d \textbf{y} / dt = \textbf{C} \, d \textbf{x} / dt = \textbf{C} \textbf{A} \textbf{x}$, the initial amplification rate of perturbations to a stable steady state can be written as

2 $$\begin{aligned} \left. \frac{d \vert \vert \textbf{y} \vert \vert }{dt} \right| _{t = 0} = \frac{\mathbf {x_0}^T \left( \textbf{C}^T \textbf{C} \textbf{A} + \textbf{A}^T \textbf{C}^T \textbf{C} \right) \mathbf {x_0}}{2 \sqrt{\mathbf {x_0}^T \textbf{C}^T \textbf{C} \mathbf {x_0}}} = \frac{\mathbf {x_0}^T H \left( \textbf{C}^T \textbf{C} \textbf{A} \right) \mathbf {x_0}}{\sqrt{\mathbf {x_0}^T \textbf{C}^T \textbf{C} \mathbf {x_0}}} \, , \end{aligned}$$

where $H(\textbf{C}^T \textbf{C} \textbf{A}) = (\textbf{C}^T \textbf{C} \textbf{A} + \textbf{A}^T \textbf{C}^T \textbf{C}) / 2$ is the Hermitian part of $\textbf{C}^T \textbf{C} \textbf{A}$.

The dominant eigenvalue of $H(\textbf{C}^T \textbf{C} \textbf{A})$ is real because $H(\textbf{C}^T \textbf{C} \textbf{A})$ is real and symmetric. If condition (1) is satisfied, then (see eq. 2) the quadratic form $\mathbf {x_0}^T H(\textbf{C}^T \textbf{C} \textbf{A}) \mathbf {x_0}$ cannot be negative semidefinite [13]. Therefore, there must exist some perturbations $\mathbf {x_0}$ that are temporarily amplified in the system output, i.e., perturbations for which the growth rate of the output norm is positive ($\vert \vert \textbf{C} \textbf{A} \mathbf {x_0} \vert \vert > 0$, with $\vert \vert \cdot \vert \vert$ indicating the Euclidean norm of vectors). These perturbations belong to the so-called g-reactivity basin of the equilibrium [12, 14], which is defined (see again eq. 2) via the quadratic form in $\mathbb {R}^n$

$$\begin{aligned} \mathbf {x_0}^T H(\textbf{C}^T \textbf{C} \textbf{A}) \mathbf {x_0} > 0 . \end{aligned}$$

Trajectories that originate in a neighborhood of the stable (yet g-reactive) steady state within its g-reactivity basin will generate a transient epidemic wave, while perturbations that lie outside the basin will monotonically decay in the system output without producing an outbreak. Therefore, initial conditions may play a crucial role in the development of short-term epidemics. In other words, we have pointed out that, even if the whole epidemic process is subcritical, it might be locally supercritical and thus a local infection can spur a transient epidemic wave.

In epidemiology, one might be interested in perturbations of the infected compartment only, not in perturbations of the whole state of the system (involving, for example, perturbations of the number of susceptibles). In this case, the only perturbations admitted are those with $\mathbf {x_0} \in \ker (\textbf{C})^{\bot }$, where $\ker (\textbf{C})^{\bot }$ is the orthogonal complement of the kernel of $\textbf{C}$. Then, it is possible to show [12] that g-reactivity can be described by the following index, which we will term *epidemicity index*

3 $$\begin{aligned} r = \sup _{\mathbf {x_0} \ne \textbf{0}} \frac{1}{\vert \vert \mathbf {y_0} \vert \vert } \left. \frac{d \vert \vert \textbf{y} \vert \vert }{dt} \right| _{t = 0} = \lambda _{\max } (H(\textbf{C} \textbf{A} \textbf{C}^+)) . \end{aligned}$$

In Eq. (3), $H(\textbf{C} \textbf{A} \textbf{C}^+)$ is the Hermitian part of matrix $\textbf{C} \textbf{A} \textbf{C}^+$, while $\textbf{C}^+ = \textbf{C}^T ( \textbf{C} \textbf{C}^T)^{-1}$ is the right pseudo-inverse of matrix $\textbf{C}$ (note that $\textbf{C} \textbf{C}^T$ is invertible because $\textbf{C}$ is full rank). The pseudo-inverse of a matrix is a generalization of the matrix inverse. Indeed, $\textbf{C} \textbf{C}^+ = \textbf{I}$, with $\textbf{I}$ being the identity matrix, even in cases where $\textbf{C}$ is not square. Eqn. (3) can be used as a g-reactivity test instead of (1). Clearly, g-reactive equilibria are characterized by the condition $r > 0$. This can be usefully exploited if $q \ll n$, because in that case matrix $H(\textbf{C} \textbf{A} \textbf{C}^+)$ has a much smaller dimension than $H(\textbf{C}^T \textbf{C} \textbf{A})$.

#### Relation to reproduction numbers

It is interesting, especially for control purposes, to link the epidemicity index to the reproduction number $\mathcal {R}_0$. When ${\textbf{y}}$ is the vector of the states-at-infection, it is possible to compute $\mathcal {R}_0$ using the next-generation matrix (NGM) approach originally proposed by Diekmann et al. [15]. The dynamics of ${\textbf{y}}$ close to DFE is described by a reduced Jacobian

$$\begin{aligned} \frac{d \textbf{y} }{dt} = {\textbf{J}} {\textbf{y}} = ({\textbf{T}} + {\mathbf {\Sigma }}) {\textbf{y}} , \end{aligned}$$

where ${\textbf{T}}$ is the transmission matrix and ${\mathbf {\Sigma }}$ the transition matrix. As shown in [15], $\mathcal {R}_0$ is the spectral radius $\rho$ of the NGM, indicated with $\textbf{K}$ and defined as:

$$\begin{aligned} \textbf{K} = -{\textbf{T}} {\mathbf {\Sigma }}^{-1} . \end{aligned}$$

The disease can become endemic if $\mathcal {R}_0 = \rho (\textbf{K}) > 1$. Since the components of ${\textbf{y}}$ are the states-at-infection, we can choose the matrix ${\textbf{C}}$ in (1) to be the identity matrix. Thus the epidemicity index *r* is the dominant eigenvalue of the Hermitian part of the matrix ${\textbf{T}} + {\mathbf {\Sigma }}$, corresponding to a reduced-order Jacobian describing the linearized dynamics of the infection subsystem. The most interesting case we want to explore is the one in which a subthreshold epidemic might occur: the DFE is stable ($\mathcal {R}_0<1$), but g-reactive (i.e, the epidemicity index is positive, $r>0$). These two conditions can be rewritten in terms of matrices ${\textbf{T}}$ and ${\mathbf {\Sigma }}$ as

4 $$\begin{aligned} \rho (-{\textbf{T}} {\mathbf {\Sigma }}^{-1}) < 1 \quad \text {and} \quad \lambda _{\max }(H({\textbf{T}}+{\mathbf {\Sigma }})) > 0 . \end{aligned}$$

When containment measures are enforced and the prevalence of susceptibles changes over time *t*, it is possible to write (4) using the *effective* indices $\mathcal {R}_t = \rho (-{\textbf{T}}_t {\mathbf {\Sigma }}_t^{-1})$ and $r_t =\lambda _{\max } (H({\textbf{T}}_t+{\mathbf {\Sigma }}_t))$. These may be useful to study the possible revamping of a declining epidemic outbreak.

#### Choice of a suitable norm to measure transient outbreaks

Although the Euclidean $\ell ^2$-norm was originally proposed to measure transient amplification in discrete-time systems [16], as also typically done in reactivity analysis of continuous-time applications [1, 12], other norms can be used as well [17]. In particular, for epidemiological and disease ecology applications, the $\ell ^1$-norm might be a more appropriate choice, especially for trajectories departing from the DFE, for which only positive perturbations of the infection subsystem are admissible. The $\ell ^1$-norm measures the maximum transient growth of a perturbation based on the absolute deviation from the DFE. As such, it has a more straightforward biological interpretation compared to the $\ell ^2$-norm, which is instead based on Euclidean distances. For instance, if ${\textbf{y}} (t)$ is the vector whose components are the numbers $I_j(t)$ of infected people of age of infection *j* (with $j = 1,2,\dots ,q$) at time *t*, then the $\ell ^1$-norm $\vert \vert \textbf{y}(t) \vert \vert _1 = \sum _{j=1}^q \vert I_j(t) \vert = \sum _{j=1}^q I_j(t)$ is the total number of infected individuals at time *t*.

#### Discrete-time dynamics

It is easy to extend our approach to discrete-time epidemiological models. This kind of models arises as a computationally useful discretization of an intrinsically continuous process, described, e.g., by a set of integro-differential equations. Also, in this way it is possible to incorporate the typically available epidemiological data, in which the number of infected people is reported, for instance, daily or weekly. In this case, the linearized dynamics of the vector ${\textbf{y}}$ at discrete time *k*, with $k = 1, 2, \ldots$, is described by

$$\begin{aligned} {\textbf{y}} (k+1) = {\textbf{J}} {\textbf{y}} (k) = ({\textbf{T}} + {\mathbf {\Sigma }}) {\textbf{y}}(k) . \end{aligned}$$

where again ${\textbf{T}}$ is the transmission matrix and ${\mathbf {\Sigma }}$ the transition matrix. For discrete-time systems, reactivity has been defined as (the natural logarithm of) the maximum growth achieved in one step by any perturbation to an asymptotically stable steady state [16]: if at least some perturbations can be initially amplified, the equilibrium is defined as reactive. Epidemicity thus measures the finite growth rate of the fastest-growing perturbation to an asymptotically stable DFE [5, 11, 14, 18, 19]. Also, by analogy with the definition of $\mathcal {R}_0$ in the continuous-time case, Allen and van den Driessche [20] proved that the RN coincides with the spectral radius of the NGM $\textbf{V} = \textbf{T} (\textbf{I} - \mathbf {\Sigma })^{-1}$.

In discrete-time systems, we then define our epidemicity index $\mathcal {E}$ as the maximum growth, evaluated in the $\ell ^1$-norm, achieved in one step by any perturbation of the current state of the system. Therefore, we have

5 $$\begin{aligned} \mathcal {E} = \max _{\vert \vert \textbf{y}(0) \vert \vert _1 \ne 0} \frac{\vert \vert \textbf{y}(1) \vert \vert _1}{\vert \vert \textbf{y}(0) \vert \vert _1} = \max _{\vert \vert \textbf{y}(0) \vert \vert _1 \ne 0} \frac{\vert \vert \textbf{J} \textbf{y}(0) \vert \vert _1}{\vert \vert \textbf{y}(0) \vert \vert _1} = \max _{1 \le j \le q} \sum _{z = 1}^q \vert J_{zj} \vert , \end{aligned}$$

where $J_{zj}$ is the element at the *z*-th row and *j*-th column of the Jacobian matrix $\textbf{J}$. The sufficient condition to avoid transient outbreaks is thus $\mathcal {E} < 1$. Therefore, we may have recurrence of subthreshold epidemics when $\rho (\textbf{V}) <1$ and $\mathcal {E} > 1$.

### Spatial models and the topology of networks

Disease spread takes place in both time and space. It is thus important to provide suitable descriptions of the spatial structure of epidemiological models. This structure can be described at various resolutions, ranging from entire countries [21] to individual households [22]. A commonly used mathematical framework for describing a wide range of spatial epidemiological models is a so-called metacommunity network model. In this framework, local communities—possibly comprising compartments for the populations of hosts, disease vectors, and pathogens—are represented as spatial nodes connected by graph links.

Waterborne pathogens can spread through waterways. Models of such diseases typically incorporate the fluvial network as a potential connection among local communities [23]. Fluvial networks resemble tree-like graphs, where each node represents a local community and is connected to a few other nodes, corresponding to the communities immediately upstream and downstream. The graph links are directed, as the flow direction influences the probability of movement of the pathogen. An ideal model to represent fluvial networks is the optimal channel network (OCN), i.e., a mathematical structure characterized by scaling forms that closely conform to the observed geomorphological features of real river networks [24–27]. Because of this feature, OCNs have often been used as a template for the structure of the landscape in epidemiological applications [28–33]. OCNs typically are randomly generated following the algorithm described in Bertuzzo et al. [34] and Bertuzzo et al. [35], based on the procedure proposed by Rigon et al. [36].

In addition to the spread of pathogens along the river network, human mobility—of both susceptible and infected hosts—can further amplify the spread of the disease. Epidemiological models can therefore account for multiple layers of network connectivity (e.g., fluvial networks and human mobility), resulting in what are known as multiplex networks [37]. The human mobility network is typically represented as a complete graph, where each node is connected to all others. The weight of each link represents the intensity of mobility flux, which can be estimated using data sources such as census data on commuting travels [38], commercial flight data [39], mobile phone data [40, 41], or predicted through mathematical models of human mobility, such as the gravity model [42] or the radiation model [43].

For airborne diseases, human mobility and social contacts are the primary drivers of spatial spread. In such cases, mathematical models can also employ a multiplex framework to describe the human mobility network, so as to account for various types of movements, including, for instance, work commuting, recreational activities, long-distance travel, and immigration flows.

## Conditions for transient epidemics in spatially-explicit compartmental models of water-related diseases

Water-related diseases encompass a broad spectrum of pathologies that are linked to water either because contaminated water is used for drinking or preparing food (e.g. cholera, typhoid), or because water bodies are the habitat of vectors transmitting the disease (e.g., malaria, dengue, schistosomiasis). The related burden of disease is quite high. For instance, unsafe water sources are responsible for 1.4 million deaths each year [44]. Understanding the ecology of pathogens and vectors is fundamental to describing the dynamics of the diseases via appropriate modelling, which also accounts for the hydrology of water bodies. In particular, we will focus our attention on waterborne diseases, namely those pathologies that are linked to the ingestion of water contaminated by human or animal faeces or urine containing bacteria, viruses or parasites.

### The basic model

Waterborne pathogen outbreaks can be described by a network model [45] that has already been used as starting point for both theoretical studies on waterborne disease dynamics [30–32] and the analysis of real-world cholera epidemics [19, 23, 46–52]. The model describes local epidemiological, demographic, and ecological processes, as well as the effects of pathogen transport along water systems and short-term human mobility on disease propagation. The network nodes represent *n* human communities of assigned population size, arranged in a given spatial setting, and connected by hydrologic pathways and human mobility.

Let $S_i(t)$ and $I_i(t)$ be the local abundances of susceptible and infected individuals in each node *i* of the network at time *t*, and let $B_i(t)$ be the concentration of pathogens in the local water reservoirs which human communities have access to. Epidemiological dynamics and pathogen transport over the hydrologic and human mobility networks can be described by the following set of 3*n* ordinary differential equations:

6 $$\begin{aligned} \frac{dS_i}{dt}&= \mu (N_i - S_i) - \left[ \left( 1 - m^S_i \right) \beta _i \frac{B_i}{K + B_i} + m^S_i \sum _{j=1}^n Q_{ij} \beta _j \frac{B_j}{K + B_j} \right] S_i \nonumber \\ \frac{dI_i}{dt}&= \left[ \left( 1 - m^S_i \right) \beta _i \frac{B_i}{K + B_i} + m^S_i \sum _{j=1}^n Q_{ij} \beta _j \frac{B_j}{K + B_j} \right] S_i - \left( \mu + \delta + \gamma \right) I_i \nonumber \\ \frac{dB_i}{dt}&= - (\nu _i + l_i) B_i + \frac{1}{W_i} \sum _{j=1}^n l_j P_{ji} W_j B_j + \frac{p_i}{W_i} \left[ \left( 1 - m^I_i \right) I_i + \sum _{j=1}^n m^I_j Q_{ji} I_j \right] . \end{aligned}$$

As for the human host population, the dynamics of the susceptible compartment in each community (first equation of model (6)) is described as a balance between population demography and infections due to exposure to the pathogen. The host population, if uninfected, is assumed to be at demographic equilibrium $N_i$, with $\mu$ being the human mortality rate. The parameter $\beta _i$ represents the site-specific rate of exposure to contaminated water, and $B_i / (K + B_i)$ is the dose-response function describing the probability of becoming infected due to the exposure to a concentration $B_i$ of pathogens (with *K* being the half-saturation constant in the sense of Codeço [53]). Exposure to contaminated water for susceptible people of community *i* can occur either in their home community (with probability $1 - m^S_i$, with $m^S_i$ being the overall probability of exposure outside the home site *i*, as determined by the mobility of susceptible individuals) or elsewhere (with probability $m^S_i Q_{ij}$, with $Q_{ij}$ representing the probability that water contacts taking place outside the home site *i* occur in site $j \ne i$, $Q_{ii} = 0$). Other routes of infection, such as fast human-to-human transmission, which have been proposed for waterborne diseases like cholera, are here neglected for simplicity, but could be dealt with within the same modeling framework [54].

The temporal evolution of the infected compartment (second equation of model (6)) is a balance between newly infected individuals and losses due to recovery or natural/pathogen-induced mortality, with $\delta$ and $\gamma$ being the rates of disease-induced mortality and recovery from infection, respectively. Note that the recovered compartment is not modeled explicitly, so that individuals who recover from the acute phase of disease are simply removed from the population, as they were conferred life-long immunity to reinfection. For cholera, as an example, recent estimates place the duration of immunity in the range 2.3-$-$3.0 years [52]. As such, loss of acquired immunity is unlikely to influence transient, short-term epidemic dynamics, which are the main focus of the present work. Although simplistic, the choice of neglecting the dynamics of recovered individuals can be deemed reasonable for the problem at hand. Extensions are, however, possible to account for processes like vaccination or immunity loss, as we will see in the section devoted to the analysis of the cholera epidemic in Haiti, which has been characterized by periodic revamping.

As for the pathogen population, the dynamics of the local concentrations of pathogens in the aquatic environment (third equation of model (6)) is given by a balance between water contamination, pathogen mortality, and hydrologic transport. Pathogens are released into the water (i.e., excreted) by infected individuals (from either the local community, with probability $1 - m^I_i$, with $m^I_i$ being the overall probability of contamination outside the home site *i*, as determined by the mobility of infected individuals; or elsewhere, with probability $m^I_j Q_{ji}$) at a site-specific rate $p_i$ and immediately diluted in a well-mixed local water reservoir of size $W_i$. Free-living pathogens are assumed to die at rate $\nu _i$. They can also move between any two neighboring nodes of the hydrologic network (say, from *i* to *j*) at rate $l_i$ and with probability $P_{ij}$.

Some of the parameters of model (6)—namely those related to human demography ($\mu$) and the physiological response to the disease ($\delta$, $\gamma$, *K*) – are assumed to be constant over the spatial scales considered in this study. All the other parameters are allowed to be possibly site-dependent. A summary of the state variables and parameters of model (6) is given in Table 1.

**Table 1:** State variables and parameters of model (6), and parameters of the output transformation (7)

Symbol	Definition
	*State variables*
$S_i$	Abundance of susceptible human hosts at site *i* ($i = 1 \ldots n$)
$I_i$	Abundance of infected human hosts at site *i*
$B_i$	Concentration of pathogens in the water reservoirs of site *i*
	*Parameters – local processes*
$N_i$	Human population size in absence of disease at site *i*
$\mu$	Baseline human mortality rate (site-independent)
$\beta _i$	Rate of exposure to contaminated water at site *i*
*K*	Half-saturation constant of dose-response function (site-independent)
$\delta$	Disease-induced mortality rate (site-independent)
$\gamma$	Recovery rate (site-independent)
$p_i$ [$\theta _i$]	Rate of water contamination at site *i* [$\theta _i = p_i / K$, rescaled contamination rate]
$W_i$	Size of the water reservoir of site *i*
$\nu _i$	Pathogen mortality rate at site *i*
	*Parameters – spatial coupling mechanisms*
$m_i^S$	Overall probability of exposure outside home site *i*
$m_i^I$	Overall probability of contamination outside home site *i*
$Q_{ij}$	Probability of water contact at *j* conditional to occurring outside home site *i*
$l_i$	Hydrologic transport rate of pathogens at site *i*
$P_{ij}$	Probability of hydrologic pathogen transport between sites *i* and *j*
	*Parameters – output transformation*
${c_I}_i$	Weight assigned to infected hosts at site *i*
${c_B}_i$	Weight assigned to bacterial concentration at site *i*

### Analytical results

To study short-term pathogen outbreaks, we seek for conditions under which small perturbations to the DFE, the state of model (6) in which $S_i = N_i$, $I_i = 0$ and $B_i = 0$ for all *i*’s, can initially grow [5]. The dynamics of model (6)) in a neighborhood of the DFE are determined by the Jacobian matrix

$$ {\mathbf {J_0}} = \begin{bmatrix} - \mu {\mathbf {U_n}} &  {\mathbf {0_n}} &  - \left[ \left( {\mathbf {U_n}} - {\mathbf {m^S}} \right) {\textbf{N}} + {\mathbf {m^S}} {\textbf{N}} {\textbf{Q}} \right] {\mathbf {\beta }} \\ {\mathbf {0_n}} &  -\phi {\mathbf {U_n}} &  \left[ \left( {\mathbf {U_n}} - {\mathbf {m^S}} \right) {\textbf{N}} + {\mathbf {m^S}} {\textbf{N}} {\textbf{Q}} \right] {\mathbf {\beta }} \\ {\mathbf {0_n}} &  {\mathbf {\theta }} {\textbf{W}}^{-1} \left( {\mathbf {U_n}} - {\mathbf {m^I}} + {\textbf{Q}}^T {\mathbf {m^I}} \right) &  - {\mathbf {\nu }} - \left( {\mathbf {U_n}} - {\textbf{W}}^{-1} {\textbf{P}}^T {\textbf{W}} \right) {\textbf{l}} \end{bmatrix}, $$

where: ${\mathbf {U_n}}$ is the identity matrix of dimension *n*; $\phi = \mu + \delta + \gamma$; and ${\textbf{N}}$, ${\textbf{W}}$, ${\mathbf {\beta }}$, ${\mathbf {\theta }}$, ${\mathbf {\nu }}$, ${\mathbf {m^S}}$, ${\mathbf {m^I}}$, ${\textbf{l}}$ are diagonal matrices with positive entries corresponding to the parameters $N_i$, $W_i$, $\beta _i$, $\theta _i = p_i / K$, $\nu _i$, $m^S_i$, $m^I_i$, and $l_i$ (with $i = 1, \cdots , n$).

According to what stated in the previous section, the DFE of model (6) is g-reactive if there exist small perturbations that can lead to a transient growth of the Euclidean norm of a suitable system output (${\textbf{y}}$) that is linked to the full state of the system, ${\textbf{x}} = [{\textbf{s}}^T, {\textbf{i}}^T, {\textbf{b}}^T]^T$. The latter is a 3*n*-dimensional vector whose components ${\textbf{s}} = [S_1, \cdots , S_n]^T$, ${\textbf{i}} = [I_1, \cdots , I_n]^T$ and ${\textbf{b}} = [B_i / K, \cdots , B_n / K]^T$ correspond to susceptible humans, infected humans and bacterial concentrations (properly rescaled). The output is defined by a linear transformation ${\textbf{y}} = {\textbf{C}} {\textbf{x}}$, where ${\textbf{C}}$ is a full-rank $q \times 3n$ real matrix, $q \le 3n$; hence ${\textbf{y}}$ is a *q*-dimensional vector. Here, we define matrix ${\textbf{C}}$ as

7 $$\begin{aligned} {\textbf{C}} = \begin{bmatrix} {\mathbf {0_n}} &  {\mathbf {c_I}} &  {\mathbf {0_n}} \\ {\mathbf {0_n}} &  {\mathbf {0_n}} &  {\mathbf {c_B}} \end{bmatrix} , \end{aligned}$$

where ${\mathbf {0_n}}$ is the null matrix of dimension *n*, while ${\mathbf {c_I}}$ and ${\mathbf {c_B}}$ are diagonal matrices with positive elements representing the weights given to the infected and bacterial components of the state space in the output transformation, respectively. Thus, we have ${\textbf{y}} = [{c_I}_1 I_1, \cdots , {c_I}_n I_n, {c_B}_1 B_1 / K, \cdots , {c_B}_n B_n / K]^T$. Note that temporary fluctuations of the susceptible compartment cannot directly influence the g-reactivity properties of the DFE, because susceptible human hosts do not contribute to the system output.

Given the output transformation matrix ${\textbf{C}}$ from (7), the DFE of model (6) is g-reactive ($\ell ^2$-norm) if

8 $$\begin{aligned} \lambda _{\max } ({\mathbf {H_0}}) > 0 , \end{aligned}$$

where $\lambda _{\max } ({\mathbf {H_0}})$ indicates the dominant eigenvalue of matrix ${\mathbf {H_0}} = H( {\textbf{C}} {\mathbf {J_0}} {\textbf{C}}^+ ) = \frac{1}{2} ({\textbf{C}} {\mathbf {J_0}} {\textbf{C}}^+ + ({\textbf{C}}^+)^T {\mathbf {J_0}}^T {\textbf{C}}^T)$, that is the Hermitian part of matrix ${\textbf{C}} {\mathbf {J_0}} {\textbf{C}}^+$, with ${\textbf{C}}^+ = {\textbf{C}}^T ( {\textbf{C}} {\textbf{C}}^T)^{-1}$ being the right pseudo-inverse of matrix ${\textbf{C}}$ and ${\mathbf {J_0}}$ the Jacobian evaluated at the DFE. Inequality (8) represents the necessary – yet not sufficient – condition for the occurrence of a (short-term) epidemic outbreak. In fact, transient waves of infections may actually occur only for perturbations lying within the g-reactivity basin of the DFE.

It is also worth noticing that the g-reactivity properties of the DFE can actually be evaluated, as shown by Mari et al. [5], based on a matrix of reduced order *n*, namely ${\textbf{F}}_0 = {\mathbf {T_0}} + {\mathbf {E_0}} + {\mathbf {E_0^S}} + {\mathbf {E_0^I}} + {\mathbf {E_0^{SI}}}$, where

$$\begin{aligned} {\mathbf {T_0}}&= {\mathbf {\nu }}^{-1} \left( {\mathbf {c_B}} {\textbf{W}}^{-1} {\textbf{P}}^T {\textbf{W}} {\mathbf {c_B}}^{-1} - {\mathbf {U_n}} \right) {\textbf{l}} ,\\ {\mathbf {E_0}}&= \frac{{\mathbf {\nu }}^{-1}}{4 \phi } \left[ {\mathbf {c_I}} \left( {\mathbf {U_n}} - {\mathbf {m^S}} \right) {\textbf{N}} {\mathbf {\beta }} {\mathbf {c_B}}^{-1} + {\mathbf {c_B}} {\mathbf {\theta }} {\textbf{W}}^{-1} \left( {\mathbf {U_n}} - {\mathbf {m^I}} \right) {\mathbf {c_I}}^{-1} \right] ^2 , \\ {\mathbf {E_0^{SI}}}&= \frac{{\mathbf {\nu }}^{-1}}{4 \phi } \left[ {\mathbf {c_I}} {\mathbf {m^S}} {\textbf{N}} {\textbf{Q}} {\mathbf {\beta }} {\mathbf {c_B}}^{-1} + {\mathbf {c_B}} {\mathbf {\theta }} {\textbf{W}}^{-1} {\textbf{Q}}^T {\mathbf {m^I}} {\mathbf {c_I}}^{-1} \right] ^2 , \\ {\mathbf {E_0^S}}&= \frac{{\mathbf {\nu }}^{-1}}{2 \phi } \left[ {\mathbf {c_I}} \left( {\mathbf {U_n}} - {\mathbf {m^S}} \right) {\textbf{N}} {\mathbf {\beta }} {\mathbf {c_B}}^{-1} + {\mathbf {c_B}} {\mathbf {\theta }} {\textbf{W}}^{-1} \left( {\mathbf {U_n}} - {\mathbf {m^I}} \right) {\mathbf {c_I}}^{-1} \right] {\mathbf {c_I}} {\mathbf {m^S}} {\textbf{N}} {\textbf{Q}} {\mathbf {\beta }} {\mathbf {c_B}}^{-1} , \textrm{and} \\ {\mathbf {E_0^I}}&= \frac{{\mathbf {\nu }}^{-1}}{2 \phi } \left[ {\mathbf {c_I}} \left( {\mathbf {U_n}} - {\mathbf {m^S}} \right) {\textbf{N}} {\mathbf {\beta }} {\mathbf {c_B}}^{-1} + {\mathbf {c_B}} {\mathbf {\theta }} {\textbf{W}}^{-1} \left( {\mathbf {U_n}} - {\mathbf {m^I}} \right) {\mathbf {c_I}}^{-1} \right] {\mathbf {c_B}} {\mathbf {\theta }} {\textbf{W}}^{-1} {\textbf{Q}}^T {\mathbf {m^I}} {\mathbf {c_I}}^{-1} \end{aligned}$$

correspond to the contribution of hydrologic pathogen transport (${\mathbf {T_0}}$), locally occurring exposure and contamination (${\mathbf {E_0}}$), mobility-driven exposure and contamination (${\mathbf {E_0^{SI}}}$), and a mixture of local and mobility-driven processes (${\mathbf {E_0^S}}$ and ${\mathbf {E_0^I}}$). The transient epidemicity condition can thus equivalently be stated [5] as

9 $$\begin{aligned} \mathcal {E}_0 = \lambda _{\max } ({\mathbf {F_0}}) > 1 . \end{aligned}$$

Condition (9) parallels the asymptotic stability criterion based on the generalized $\mathcal {R}_0$, according to which the DFE of model (6) is unstable (a necessary condition for long-term pathogen invasion and endemic disease transmission) if

10 $$\begin{aligned} \mathcal {R}_0 = \lambda _{\max } ({\mathbf {G_0}}) > 1 , \end{aligned}$$

where ${\mathbf {G_0}}$ is the generalized reproduction matrix [31, 46] defined as ${\mathbf {G_0}} = {\mathbf {T_0'}} + {\mathbf {R_0}} + {\mathbf {R_0^S}} + {\mathbf {R_0^I}} + {\mathbf {R_0^{SI}}}$ [5], with

$$\begin{aligned} {\mathbf {T_0'}}&= {\mathbf {\nu }}^{-1} \left( {\textbf{W}}^{-1} {\textbf{P}}^T {\textbf{W}} - {\mathbf {U_n}} \right) {\textbf{l}} ,\\ {\mathbf {R_0}}&= {\mathbf {\nu }}^{-1} {\mathbf {\theta }} {\textbf{W}}^{-1} \left( {\mathbf {U_n}} - {\mathbf {m^I}} \right) \left( {\mathbf {U_n}} - {\mathbf {m^S}} \right) {\textbf{N}} {\mathbf {\beta }} ,\\ {\mathbf {R_0^S}}&= {\mathbf {\nu }}^{-1} {\mathbf {\theta }} {\textbf{W}}^{-1} \left( {\mathbf {U_n}} - {\mathbf {m^I}} \right) {\mathbf {m^S}} {\textbf{N}} {\textbf{Q}} {\mathbf {\beta }} ,\\ {\mathbf {R_0^I}}&= {\mathbf {\nu }}^{-1} {\mathbf {\theta }} {\textbf{W}}^{-1} {\textbf{Q}}^T {\mathbf {m^I}} \left( {\mathbf {U_n}} - {\mathbf {m^S}} \right) {\textbf{N}} {\mathbf {\beta }} , \textrm{and} \\ {\mathbf {R_0^{SI}}}&= {\mathbf {\nu }}^{-1} {\mathbf {\theta }} {\textbf{W}}^{-1} {\textbf{Q}}^T {\mathbf {m^I}} {\mathbf {m^S}} {\textbf{N}} {\textbf{Q}} {\mathbf {\beta }} . \end{aligned}$$

Taken together, conditions (9) and (10) generalize g-reactivity and stability analysis results found in spatially-implicit applications [11] to spatial hydrological settings of any complexity.

### Application to realistic hydrological networks

**Fig. 1:**
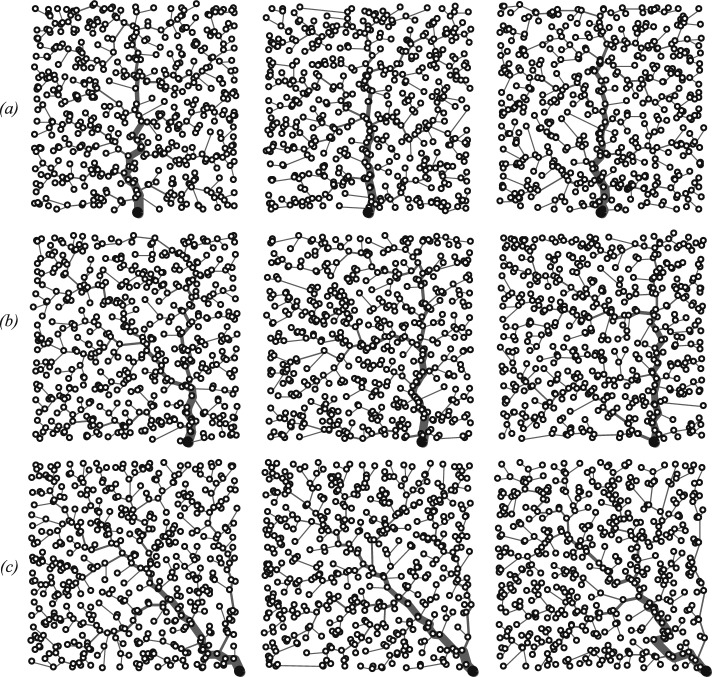
Examples of OCN topologies characterized by different positions of the outlet. The network outlet (larger black node) is located **a** at the mid-point of the bottom side of the domain, **b** half-way between the mid-point and the right end-point of the bottom side of the domain, or **c** at the right end-point of the bottom side of the domain. Three different replicas (out of 16 considered overall, see text) are representatively shown for each of the three topologies. Figure after Mari et al. [5] (color figure online)

To analyze outbreak/establishment conditions in a realistic setting, here we assume that human communities constitute the nodes of an OCN landscape embedded in a square of unitary side (this condition can be relaxed by imposing periodic boundary conditions). We consider OCNs endowed with three different spatial configurations, characterized by the position of the outlet (Fig. 1). Since the generation of OCNs is an intrinsically stochastic process [34, 35], we consider several (16) replicas for each network geometry. The total number of network nodes ($n \approx 500$) is preserved in each geometry and replica.

As for water-mediated pathogen movement, we apply conservative transport everywhere except for the outlet, from which pathogens are removed from the network. The specification of matrix ${\textbf{P}} = [P_{ij}]$, describing the probability for the movement of pathogens from node *i* to node *j* (see (6)), thus goes as follows. Let $p^d$ be the probability of downstream transport (and $1 - p^d$ the probability of upstream transport). If *h* is a headwater node and *j* its downstream neighbor, $P_{hj} = 1$ (reflecting boundary); for the inner nodes *i* of the network, $P_{ij} = p^d$ if *j* is the downstream neighbor, or $P_{ij} = (1 - p^d)/n^u_i$ if *j* is one of the $n^u_i$ upstream neighbors of node *i*, with $n^u_i = 2$ for most *i*’s; at the outlet, *o*, pathogens are discharged from the network with probability $p^d$ (absorbing boundary), while $P_{oj} = (1 - p^d)/n^u_o$ if *j* is one of the $n^u_o$ upstream neighbors of node *o*. Note that absorbing conditions prevent pathogens from accumulating at the network outlet. The parameters of the hydrological connectivity network are summarized in Table 2.

**Table 2:** Parameters of the hydrological connectivity and human mobility networks

Symbol	Definition
	*Hydrological connectivity*
${\textbf{P}} = [P_{ij}]$	Hydrological connectivity network
$p^d$	Probability of downstream transport
$n_i^u$	Number of upstream nodes
	*Human mobility*
${\textbf{Q}} = [Q_{ij}]$	Human mobility matrix
$d_{ij}$	Euclidean distance between nodes *i* and *j*
*D*	Distance scaling factor

On top of hydrologic connectivity, a second mobility layer accounting for human movement must also be specified in model (6). Pairwise movement probabilities $Q_{ij}$ are described through a gravity model [42, 55] in which the attractiveness of node *j* for node *i* is assumed to be directly proportional to the population size of *j* and inversely proportional to the distance $d_{ij}$ between the two nodes (through an exponential kernel with scale factor *D*), i.e., $Q_{ij} \propto N_j \exp (-d_{ij}/D)$ (if $i \ne j$, $Q_{ii} = 0$). Movement probabilities $Q_{ij}$ constitute the entries of the human mobility matrix (${\textbf{Q}} = [Q_{ij}]$). To stipulate that ${\textbf{Q}}$ is row-stochastic (i.e., a matrix in which rows sum up to one), outgoing mobility fluxes are normalized by $\sum _{k \ne i}^n N_k \exp (-d_{ik}/D)$. Different mobility models can be easily accommodated in the formalism of system (6), provided that human mobility can be expressed in terms of movement probability (as quantified by $m^S_i$ and $m^I_i$) and trip distribution (e.g., in the form of an origin–destination matrix, as quantified by ${\textbf{Q}}$). See again Table 2 for a summary of the human mobility network parameters.

### Numerical analysis of threshold conditions

**Fig. 2:**
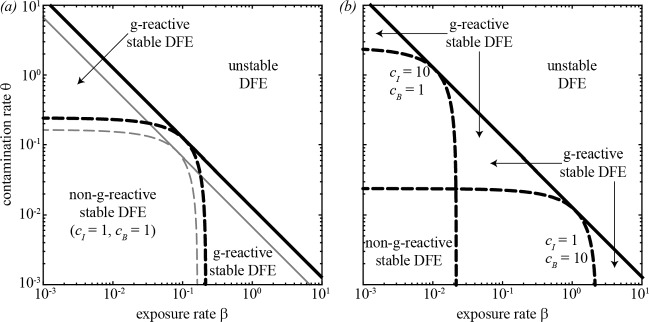
Stability and epidemicity analysis for the DFE of model (6). (*a*) The solid black line ($\mathcal {R}_0 = 1$) separates the parameter combinations for which the DFE is stable ($\mathcal {R}_0 < 1$) from those for which the DFE is unstable ($\mathcal {R}_0 > 1$), while the dashed black line ($\mathcal {E}_0 = 1$) marks the boundary between the parameter sets for which the DFE is g-reactive ($\mathcal {E}_0 > 1$, for which short-term epidemic outbreaks are possible) and those for which the DFE is non-g-reactive ($\mathcal {E}_0 < 1$, for which transient epidemics are not allowed). These stability and g-reactivity thresholds refer to the leftmost OCN shown in Fig. 1*b*, but similar results were obtained with all of the other OCNs tested. The stability/g-reactivity boundaries for a local model (i.e., a model describing a set of disconnected local communities obtained from (6) by setting $l = 0$ and $m^S = m^I = 0$) are shown as thin gray lines: specifically, the local DFE is unstable above the solid line and non-g-reactive below the dashed line. (*b*) Epidemicity analysis for different choices of the entries of matrix ${\textbf{C}}$ (Eq. (7)). Parameter values [23]: $\mu = 4.2 \cdot 10^{-5}$, $\delta = 4.0 \cdot 10^{-4}$, $\gamma = 1/5$, $\nu = 1/30$, $l = 1/3$ (all rates in [day$^{-1}$]), $p^d = 0.8$, $m^S = 0.2$, $m^I = 0.05$, $D = 0.05$. All parameters are assumed to be spatially homogeneous, including the size of local communities ($N_i = N = 1$), the distribution of water resources ($W_i = W = 1$), and the weights of infected humans and bacterial concentrations in the output transformation (${c_I}_i = c_I$ and ${c_B}_i = c_B$, respectively). See Table 1 for a summary of the model parameters. Figure after Mari et al. [5] (color figure online)

Figure 2*a* shows the epidemicity and stability properties of the DFE of model (6) as a function of the human exposure and contamination rates ($\beta$ and $\theta = p / K$, respectively) when all model parameters, including the weights given to infected prevalence and bacterial abundance in the different communities, are assumed to be homogeneously distributed in space (therefore, ${\textbf{N}} = N {\mathbf {U_n}}$, ${\textbf{W}} = W {\mathbf {U_n}}$, ${\mathbf {\beta }} = \beta {\mathbf {U_n}}$, ${\mathbf {\theta }} = \theta {\mathbf {U_n}}$, ${\mathbf {\nu }} = \nu {\mathbf {U_n}}$, ${\mathbf {m^S}} = m^S {\mathbf {U_n}}$, ${\mathbf {m^I}} = m^I {\mathbf {U_n}}$, ${\textbf{l}} = l {\mathbf {U_n}}$, ${\mathbf {c_I}} = c_I {\mathbf {U_n}}$ and ${\mathbf {c_B}} = c_B {\mathbf {U_n}}$ are all scalar matrices). The results refer to the leftmost OCN replica shown in Fig. 1*b*, but the g-reactivity and stability thresholds evaluated with all the other OCN structures are virtually indistinguishable from those of Fig. 2*a*. This shows that, for an assigned specification of hydrologic transport and human mobility, the g-reactivity and stability properties of model (6) may be quite robust to variations of the underlying spatial domain, e.g., concerning the fine-scale structure of the OCN landscape or the position of the outlet node.

If both the exposure rate $\beta$ and the contamination rate $\theta$ are small in magnitude, the DFE is stable ($\mathcal {R}_0 < 1$) – which prevents long-term pathogen establishment – and non-g-reactive ($\mathcal {E}_0 < 1$, evaluated for $c_I = 1$ and $c_B = 1$) – which rules out even transient epidemic outbreaks in the community. The parameter regions characterized by either relatively large values of $\beta$ and small values of $\theta$, or relatively large values of $\theta$ and small values of $\beta$ correspond instead to a stable, g-reactive DFE ($\mathcal {R}_0 < 1$, $\mathcal {E}_0 > 1$). In this case, transient epidemic waves can be triggered by suitable perturbations to the DFE, provided that either exposure or contamination is sufficiently large, but long-term pathogen establishment and endemic transmission are not possible.

We also note from Fig. 2*a* that higher values of $\beta$ and $\theta$ are needed in the network model (which includes pathogen transport and human mobility) than in a spatially implicit setting (i.e., in a model with $l_i = 0$ and $m^S_i = m^I_i = 0$ for all *i*’s) for the DFE to be g-reactive/unstable. This is specifically due to the presence of an absorbing boundary for hydrologic pathogen transport, which makes this spatial coupling mechanism non-conservative. As a matter of fact, if water-mediated pathogen transport is negligible – or otherwise dominated by human mobility – a different result can be found, namely that epidemic/endemic transmission requires lower values of $\beta$ and $\theta$ in a spatially explicit (rather than in a spatially implicit) setting [31].

The definition of matrix ${\textbf{C}}$ given in (7) clearly influences the g-reactivity properties of the DFE. In fact, choosing different values for $c_I$ or $c_B$ can imply a change in the classification of the DFE from g-reactive to non-g-reactive, or vice versa (Fig. 2*b*). Therefore, g-reactivity classification is not absolute: in order to be meaningful, it requires a suitable (i.e., epidemiologically motivated) design of the output transformation.

**Fig. 3:**
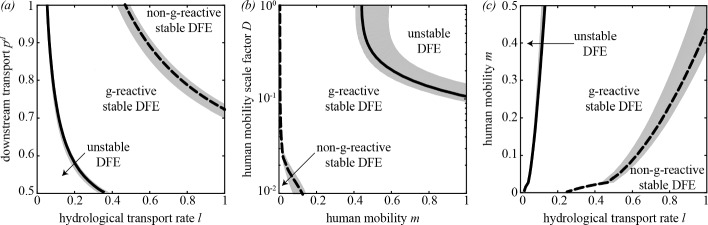
The role of spatial coupling mechanisms in disease epidemicity and endemicity. (*a*) Hydrologic transport of pathogens. (*b*) Human mobility. (*c*) Coupled effect of hydrologic pathogen transport and human mobility. Results are reported for 16 OCN replicas like those shown in Fig. 1*b*. Specifically, in each panel, the solid [dashed] black line represents the median location of the replica-specific stability ($\mathcal {R}_0 = 1$) [g-reactivity ($\mathcal {E}_0 = 1$)] boundary, while the gray-shaded areas are the envelopes of the boundaries obtained from each OCN replica. Parameter values: $\beta = 0.3$, $\theta = 0.03$, $m^S = m$, $m^I = m / 4$. Other parameters as in Fig. 2*a*. Figure after Mari et al. [5] (color figure online)

The role played by the transport/mobility parameters in triggering disease epidemicity or endemicity is shown in Fig. 3. High values of the hydrologic transport rate *l* and the downstream transport probability $p^d$ are associated with a stable, non-g-reactive DFE, while transient epidemics and endemic transmission can be found for lower values of *l* and/or $p^d$ (panel *a*). On the other hand, human mobility promotes both short-term outbreaks and long-term pathogen establishment (panel *b*). In fact, for low levels of human mobility (small $m^S$ and $m^I$) the pathogen cannot invade the system and the DFE is non-g-reactive. Finally, it is possible to study the interplay between water-mediated pathogen transport and human mobility (panel *c*). Again, high values of the hydrologic transport rate are associated with a stable, possibly non-g-reactive DFE. Conversely, high human mobility can lead to a g-reactive or unstable DFE. This general picture remains qualitatively unchanged for different values of the baseline exposure and contamination rates, although quantitative details can obviously vary. Interestingly, when looking at the parameters concerning spatial coupling mechanisms, we find that network topology may indeed influence the g-reactivity and stability properties of the DFE of model (6), especially in parameter regions characterized by relatively high hydrologic transport and/or human mobility.

## Conditions for transient epidemics in spatially-explicit models of air-borne diseases

In this section, we analyze the g-reactivity of a model for air-borne disease. We describe the spatial and temporal spread of a viral respiratory virus [56] within a set of connected communities using a susceptible-infected-removed (SIR) transmission process based on a Kermack-McKendrick-type set of integrodifferential equations with age-of-infection structure [57, 58]. To more closely resemble the discrete-time nature of surveillance data, we discretize it in time and age of infection [20], thereby obtaining an epidemiological projection matrix that can be used to simulate the transmission process. We assume, as a simplification, the knowledge of the effective reproduction number of each community at any time, i.e., we hypothesize that in real time we have an estimate of the number of secondary cases produced by one infectious case of each community. It must be remarked that in this way we implicitly incorporate the variation of the number of susceptibles into the effective RNs of the different communities.

### A discrete infection process

Consider a metacommunity composed of *N* communities connected by human mobility. We assume that the communities differ from one another because they have different effective RNs and different mobility parameters. Let the number of individuals in the infected compartment within a generic community *j* be defined as $I_j(t,\tau )$, a function of both the current time *t* and the age of infection $\tau$ [57, 58]. Also, let $C_{lj}(t)$ be the proportion of residents of community *j* who commute daily to community *l* for whatever purpose. The governing equations in continuous time give rise to the following mathematical formulation [59, 60]:

$$\begin{aligned} \dfrac{\partial I_j(t,\tau )}{\partial t} + \dfrac{\partial I_j(t,\tau )}{\partial \tau }&= -\gamma (\tau ) I_j(t,\tau ) \\ I_j(t,0)&= \displaystyle \sum _{k=1}^N z_{jk}(t) \mathcal {R}_k(t) \displaystyle \int _0^{\infty } \phi (\tau ) \ I_k(t,\tau ) \ \textrm{d}\tau \ \quad \forall t \ , \end{aligned}$$

where: $\gamma (\tau )$ represents the instantaneous rate of exit from the infected compartment (because of recovery, death, quarantine, or isolation); $\phi (\tau )$ is the rate of secondary transmission per single infectious case (per unit of RN); $\mathcal {R}_k(t)$ is the effective RN of community *k* at time *t*, namely, the number of secondary cases produced by one infectious case of community *k* at time *t* throughout the duration of the infection; and

$$\begin{aligned} z_{jk}(t) = \sum _{l=1}^N \dfrac{C_{lj}(t)n_j}{\sum _{m=1}^N C_{lm}(t) n_m} C_{lk}(t) \end{aligned}$$

accounts for each node’s exposure to infectious individuals residing in a different community, with $n_j$ and $n_m$ being the population size of nodes *j* and *m*, respectively. The terms $z_{jk}(t)$ are computed to consider all possible means of contact and infection [59, 60] by assuming homogeneous mixing of the population in the node where infection occurs. It should be noted that both $\textbf{C}$ and $\textbf{Z}$ are column-stochastic matrices, that is, $\sum _l C_{lk} = \sum _j z_{jk} = 1$. We define the probability of still being in the infected compartment at the age of infection $\tau$ as

$$\begin{aligned} p(\tau ) = \exp \left( - \int _0^{\tau } \gamma (\xi ) \textrm{d} \xi \right) . \end{aligned}$$

The probability density function of the generation times, $\beta (\tau )$, is related to this quantity via the equation

$$\begin{aligned} \beta (\tau ) = p(\tau ) \phi (\tau ) . \end{aligned}$$

One may introduce a temporal discretization of the above problem so that it can be solved computationally with a suitable time step (e.g., one day), which is practical when epidemiological data (say, drawn from daily surveillance bulletins) are used to track the spatiotemporal evolution of an outbreak and/or produce epidemiological projections. The ensuing mathematical problem has already been addressed through the use of the method of characteristics [59]. Once this problem is tackled, a process discretized in both time and age of infection is obtained, namely,

11 $$\begin{aligned} {\begin{matrix} I_{j,1}(t+1) & = \sigma _0 \displaystyle \sum _{k=1}^N z_{jk}(t) \mathcal {R}_k(t) \displaystyle \sum _{i = 1}^q f_i I_{k,i}(t) \\ I_{j,i}(t+1) & = \sigma _{i-1} I_{j,i-1}(t) \ \textrm{for } \ i \ge 2, \end{matrix}} \end{aligned}$$

where: $I_{j,i}$ is the number of infected people of infection age *i* ($i=1,\dots ,q$) in community *j* ($j=1,\dots ,N$), $f_i = \int _{i-1}^i \phi (\tau ) \textrm{d}\tau$ is a discretization of function $\phi (\tau )$ over one day, *q* is the maximum age of infection, and

$$\begin{aligned} \sigma _{i-1} = \dfrac{p(i)}{p(i-1)} \end{aligned}$$

represents the proportion of infectious individuals with age of infection $i-1$ that are still infectious on the next day.

Let us now define a column vector $\textbf{I}(t)$ that collects all the space-age of infection compartments at time *t*, that is,

$$\begin{aligned} \textbf{I}(t) = \left\{ I_{1,1}, \dots , I_{N,1}, \dots , I_{1,q}, \dots , I_{N,q}\right\} ^T . \end{aligned}$$

It is possible to rewrite equations (11) in matrix form as

$$\begin{aligned} \textbf{I}(t) = \textbf{L}_t \textbf{I}(t-1), \end{aligned}$$

where matrix $\textbf{L}_t$ is the Leslie projection matrix at time *t* [61], i.e, a block matrix taking the form

$$\begin{aligned} \textbf{L}(t) = \begin{pmatrix} \textbf{T}_{1}(t)&  \textbf{T}_{2}(t)&  \cdots &  \textbf{T}_{q-1}(t) &  \textbf{T}_{q}(t)\\ \sigma _1 \textbf{U}_{N}&  \textbf{0}_N&  \cdots &  \textbf{0}_N & \textbf{0}_N \\ \textbf{0}_N&  \sigma _2 \textbf{U}_{N}&  \cdots &  \textbf{0}_N & \textbf{0}_N \\ \vdots &  \vdots &  \ddots &  \vdots &  \vdots \\ \textbf{0}_N&  \textbf{0}_N &  \cdots &  \sigma _{q-1} \textbf{U}_{N}&  \textbf{0}_N \\ \end{pmatrix}, \end{aligned}$$

where $\textbf{U}_N$ and $\textbf{0}_N$ are the identity and null matrices of order *N*, respectively, and the submatrices $\textbf{T}_i(t)$ are defined as

12 $$\begin{aligned} \textbf{T}_i(t) = \sigma _0 f_i \begin{pmatrix} \mathcal {R}_{1}(t)z_{11}(t) & \mathcal {R}_{2}(t)z_{12}(t) &  \dots & \mathcal {R}_N(t) z_{1N}(t) \\ \mathcal {R}_{1}(t)z_{21}(t) & \mathcal {R}_{2}(t)z_{22}(t) &  \dots & \mathcal {R}_N(t) z_{2N}(t) \\ \vdots &  \vdots &  \ddots &  \vdots \\ \mathcal {R}_{1}(t)z_{N1}(t) & \mathcal {R}_{2}(t)z_{N2}(t) &  \dots & \mathcal {R}_N(t) z_{NN}(t) \\ \end{pmatrix}. \end{aligned}$$

Our reactivity analysis is based on two variables, the total abundance of infected people in a given node *j* at time *t* ($\textbf{Y}_1(t)$) and their prevalence in the population ($\textbf{Y}_2(t)$). The rationale for this choice is a better adherence to the nature of data available within real-time surveillance programs. What is usually measured is the incidence rate of newly observed cases, from which the number of infected individuals can be estimated by taking into account the recovery rate. However, the specific age of infection of the new reported cases is not usually known. Therefore, the number of infected individuals is usually estimated as a whole and not for each age of infection. Also, decisions on intervention measures are usually taken at this level of resolution. The two possible output vectors are defined as

$$\begin{aligned} \textbf{Y}_1(t) = \left\{ Y_1^1, Y_1^2, \dots , Y_1^N\right\} ^T, \textbf{Y}_2(t) = \left\{ Y_2^1, Y_2^2, \dots , Y_2^N\right\} ^T, \end{aligned}$$

where

$$\begin{aligned} Y_1^j = \displaystyle \sum _{i=1}^q I_{j,i} \ \textrm{and} \ Y_2^j = \displaystyle \dfrac{1}{n_j} \sum _{i=1}^q I_{j,i}. \end{aligned}$$

We can link the observed variables to the state variable ${\textbf{I}}(t)$ via the block matrices

$$\begin{aligned} \textbf{W}_1&= \left( \begin{array}{llll} \textbf{U}_{q}&\textbf{U}_{q}&\cdots&\textbf{U}_{q} \end{array}\right) \\ \textbf{W}_2&= \left( \begin{array}{llll} \dfrac{1}{n_1} \textbf{U}_{q}&  \dfrac{1}{n_2} \textbf{U}_{q}&  \cdots &  \dfrac{1}{n_N} \textbf{U}_{q}\\ \end{array}\right) , \end{aligned}$$

such that

$$\begin{aligned} \textbf{Y}_1(t) = \textbf{W}_1 \textbf{I}(t) \ \textrm{and} \ \textbf{Y}_2(t) = \textbf{W}_2 \textbf{I}(t). \end{aligned}$$

Both $\textbf{W}_1$ and $\textbf{W}_2$ are full-rank matrices, a property which is a necessary condition to carry out our short-term reactivity analysis on the observed variables $\textbf{Y}_1(t)$ and $\textbf{Y}_2(t)$.

### The discrete epidemicity index

The evaluation of the Leslie projection matrix on a given day allows one to carry out a quantitative analysis of short-term epidemiological dynamics. This will be done by utilizing the $l^1$ algebraic norm as stated above but the analysis can be extended to the $l^2$ norm as reported by Trevisin et al. [60].

Applying this norm to a non-negative vector $\textbf{Y}(t)$ corresponds to evaluating the sum of the vector components at time *t*, that is,

$$\begin{aligned} ||\textbf{Y}(t)||_1 = \sum _j Y_{j}(t). \end{aligned}$$

It thus follows that

$$\begin{aligned} ||\textbf{W}_1 \textbf{I}(t)||_1 = \displaystyle \sum _{j=1}^N \displaystyle \sum _{i=1}^q I_{j,i}(t) \end{aligned}$$

is the total abundance of infectious individuals in the whole metacommunity at time *t*. By recalling the column-stochasticity of matrix $\textbf{Z}$, one finds out that

$$\begin{aligned} ||\textbf{W}_1 \textbf{I}(t+1)||_1 = \displaystyle \sum _{j=1}^N \displaystyle \displaystyle \sum _{i = 1}^q \sigma _0 \mathcal {R}_j(t) f_i I_{j,i}(t) + \displaystyle \sum _{j=1}^N \displaystyle \sum _{i=1}^{q-1} \sigma _i I_{j,i}(t). \end{aligned}$$

In the $\sigma _q = 0$ limit, the above expression reduces to

$$\begin{aligned} ||\textbf{W}_1 \textbf{I}(t+1)||_1 = \displaystyle \sum _{j=1}^N \displaystyle \sum _{i = 1}^q \left( \sigma _0 \mathcal {R}_j(t) f_i + \sigma _i\right) I_{j,i}(t). \end{aligned}$$

Therefore, the problem of finding the epidemicity index $\mathcal {E}$ (Eq. (5)) is equivalent to

$$\begin{aligned} {\left\{ \begin{array}{ll} \displaystyle \max _{j,i} \displaystyle \sum _{j=1}^N \displaystyle \sum _{i = 1}^q \left( \sigma _0 \mathcal {R}_j(t) f_i + \sigma _i\right) I_{j,i}(t) \\ \mathrm {subject \ to} \ \displaystyle \sum _{j=1}^N \displaystyle \sum _{i = 1}^q I_{j,i}(t) = \ \textrm{constant}. \end{array}\right. } \end{aligned}$$

This is a linear programming (LP) problem in the variables $I_{j,i}(t)$. Since there is only one equality constraint, the optimal solution has just one basic variable, precisely the one characterized by $\max _{j,i}\left( \sigma _0 \mathcal {R}_j(t) f_i + \sigma _i\right)$ [62]. One can of course set the constant to one, so that $I_{j,i}=1$. In this case, the epidemicity index reads

13 $$\begin{aligned} \mathcal {E}(t) = \max _{j,i} \left( \sigma _0 \mathcal {R}_j(t) f_i + \sigma _i\right) . \end{aligned}$$

If we assume $\sigma _j = \sigma \ \forall j <q$ and $f_q = 0$, then

$$\begin{aligned} \mathcal {E}(t) = \sigma _0 \max _j \mathcal {R}_j(t) \max _i f_i + \sigma . \end{aligned}$$

This result shows that the maximum one-step growth of the system depends on the maximum local effective RN. In particular, the system is reactive whenever any local effective RN exceeds the threshold $(1 - \sigma ) / (\sigma _0 \max _i f_i)$ [63], which implies that there exists at least one perturbation that develops a growing response associated with a transient epidemic outbreak.

The $\ell ^1$-norm can be used also for the prevalence of infectious people. In this case, the norm is proportional to the average prevalence across the different communities. As such, a decision maker might be interested in avoiding its increase over time. The relevant algebra becomes slightly more complex. Let us consider the norm

$$\begin{aligned} ||\textbf{Y}_2(t)||_1 = ||\textbf{W}_2 \textbf{I}(t)||_1 = \displaystyle \sum _{k=1}^N \displaystyle \sum _{i=1}^q \dfrac{I_{k,i}(t)}{n_k}. \end{aligned}$$

Multiplying by the Leslie matrix, at time $t+1$ we have

$$\begin{aligned} ||\textbf{W}_2 \textbf{I}(t+1)||_1 = \sigma _0 \displaystyle \sum _{k=1}^N \displaystyle \sum _{j=1}^N z_{kj} \mathcal {R}_j(t) \dfrac{n_j}{n_k} \displaystyle \sum _{i=1}^q f_i \dfrac{I_{j,i}(t)}{n_j} + \displaystyle \sum _{k=1}^N \displaystyle \sum _{i=1}^q \sigma _i \dfrac{I_{k,i}(t)}{n_k}. \end{aligned}$$

Let us introduce the variables $y_{k,i}(t)=\dfrac{I_{k,i}(t)}{n_k}$, thus obtaining a revised form of the above formula, which now reads

$$\begin{aligned} {\begin{matrix} ||\textbf{W}_2 \textbf{I}(t+1)||_1 = \sigma _0 \displaystyle \sum _{k=1}^N \displaystyle \sum _{j=1}^N z_{kj} \mathcal {R}_j(t) \dfrac{n_j}{n_k} \displaystyle \sum _{i=1}^q f_i y_{j,i}(t) + \displaystyle \sum _{j=1}^N \displaystyle \sum _{i=1}^q \sigma _i y_{j,i}(t) = \\ = \displaystyle \sum _{j=1}^N \displaystyle \sum _{i=1}^q \left[ \left( \displaystyle \sum _{k=1}^N z_{kj} \dfrac{n_j}{n_k} \right) \mathcal {R}_j(t) f_i \sigma _0 + \sigma _i \right] y_{j,i}(t). \end{matrix}} \end{aligned}$$

Therefore, the problem of finding the epidemicity index, that is

$$\begin{aligned} \max _{||\textbf{Y}_2(t)||_1\ne 0} \dfrac{||\mathbf {Y_2(t+1)}||_1}{||\mathbf {Y_2(t)}||_1} \end{aligned}$$

is equivalent to the LP problem

$$\begin{aligned} {\left\{ \begin{array}{ll} \displaystyle \max _{j,i} \left( \sigma _0 \eta _j(t) \mathcal {R}_j(t) f_i + \sigma _i\right) y_{j,i}(t) \\ \mathrm {subject \ to} \ \displaystyle \sum _{k=1}^N \displaystyle \sum _{i = 1}^q y_{j,i}(t) = \ \textrm{constant}, \end{array}\right. } \end{aligned}$$

with $\eta _j(t) = \sum _{k=1}^N z_{kj}(t) n_j / n_k$. The solution has one basic variable, the one characterized by $\max _{j,i} \left( \sigma _0 \eta _j(t) \mathcal {R}_j(t) f_i + \sigma _i\right)$. The epidemicity index is thus given by

$$\begin{aligned} \mathcal {E}(t) = \max _{j,i} \left( \sigma _0 \eta _j(t) \mathcal {R}_j(t) f_i + \sigma _i\right) . \end{aligned}$$

It should be noted that if all $n_k$ are equal, then this solution would yield the one shown in Eq. (13). If we assume $\sigma _j = \sigma \ \forall j <q$, and $f_q=0$, then

$$\begin{aligned} \mathcal {E}(t) = \sigma _0 \max _j \eta _j(t)\mathcal {R}_j(t) \max _i f_i + \sigma . \end{aligned}$$

Therefore, if we consider disease prevalence as the output variable, the focal community might not be the one with the maximum RN, but the one with the maximum $\eta _j(t)\mathcal {R}_j(t)$, which depends on the interplay between population size, connectivity, and local RNs.

In addition, it may be of interest to consider the epidemicity subset, which is the ensemble of nodes that can produce transient responses [64]. The epidemicity subset is defined as

$$\begin{aligned} \textbf{E}(t) = \left\{ j \ \text {such that} \ \max _{||\textbf{I}_j(t)||\ne 0} \dfrac{||\textbf{W}_r\textbf{L}(t)\textbf{I}(t)||}{||\textbf{W}_r\textbf{I}(t)||} > 1\right\} . \end{aligned}$$

Its elements are defined as the nodes corresponding to

$$\begin{aligned} \sigma _0 \mathcal {R}_j(t) \max _i f_i + \sigma > 1, \end{aligned}$$

for the observed variable $\textbf{Y}_1(t)$. For $\textbf{Y}_2(t)$, instead, the nodes belonging to the epidemicity subset are those for which

$$\begin{aligned} \sigma _0 \eta _j(t)\mathcal {R}_j(t) \max _i f_i + \sigma > 1. \end{aligned}$$

If the cardinality of $\textbf{E}(t) > 1$, any perturbation to the DFE acting upon one or more of these age-space combinations would thus be at least temporarily amplified, thereby developing a transient epidemic outbreak. As an example, these perturbations may be associated with the arrival of infected individuals with specific ages of infection in specific communities of the spatial network.

### Long-term dynamics: the effective reproduction number of the system

Following the canonical NGM approach, let us decompose the Leslie projection matrix into two matrices, a transmission matrix $\textbf{T}(t)$, containing all the terms reflecting the generation of new cases (see the first equation of system (11)), and a transition matrix $\mathbf {\Sigma }(t)$, containing the transition terms (see Eq. (11)b), such that $\textbf{T}(t) + \mathbf {\Sigma }(t) = \textbf{L}(t)$. As such, the transmission matrix $\textbf{T}(t)$ is constructed as

$$\begin{aligned} \textbf{T}(t) = \begin{pmatrix} \textbf{T}_1(t)&  \textbf{0}_N&  \cdots &  \textbf{0}_N&  \textbf{0}_N\\ \textbf{0}_N&  \textbf{T}_2(t)&  \cdots &  \textbf{0}_N&  \textbf{0}_N\\ \vdots &  \vdots &  \ddots &  \vdots &  \vdots \\ \textbf{0}_N&  \textbf{0}_N&  \cdots &  \textbf{T}_{q-1}(t) &  \textbf{0}_N \\ \textbf{0}_N&  \textbf{0}_N&  \cdots &  \textbf{0}_N & \textbf{T}_q(t) \\ \end{pmatrix}, \end{aligned}$$

where matrices $\textbf{T}_i(t)$ are defined as in Eq. (12). The transition matrix, on the other hand, is defined as

$$\begin{aligned} \mathbf {\Sigma }(t) = \begin{pmatrix} \textbf{0}_N&  \textbf{0}_N&  \cdots &  \textbf{0}_N&  \textbf{0}_N\\ \sigma _1\textbf{U}_N&  \textbf{0}_N&  \cdots &  \textbf{0}_N&  \textbf{0}_N\\ \textbf{0}_N&  \sigma _2\textbf{U}_N&  \cdots &  \textbf{0}_N&  \textbf{0}_N\\ \vdots &  \vdots &  \ddots &  \vdots &  \vdots \\ \textbf{0}_N&  \textbf{0}_N&  \cdots &  \sigma _{q-1}\textbf{U}_N&  \textbf{0}_N \end{pmatrix}. \end{aligned}$$

The NGM can be built as [20, 65]

$$\begin{aligned} \textbf{K}(t) = \textbf{T}(t) \left( \textbf{U}_{N q} - \mathbf {\Sigma }(t)\right) ^{-1}, \end{aligned}$$

where $\textbf{U}_{N q}$ is the identity matrix of size $Nq \times Nq$. The effective reproduction number of the whole system, which we will from now on term global RN, to distinguish it from the effective RN’s of the different communities, is defined as the spectral radius of the NGM

$$\begin{aligned} \mathcal {R}^G(t) = \rho \left( \textbf{K}(t) \right) . \end{aligned}$$

This quantity determines the long-term behavior of the system as a whole. In a spatially connected system where local effective RNs are either above or below the unit threshold, whenever $\mathcal {R}^G(t) > 1$ there exists at least one possible perturbation of the DFE that could be indefinitely amplified over time (say, in the linearized system).

### Effect of spatial connectivity on the epidemiological indices

In the following, we shall resort to a fictitious metacommunity of the kind used in [59, 60]. To start with, we first describe the results obtained with the simplest spatial model, that is the one considering a metacommunity with just two nodes. Then, we will see how these results can be generalized to a metacommunity with any number *N* of connected local communities.

#### A two-node synthetic metacommunity

For the sake of clarity, here we analyze a simple system considering a two-node synthetic metacommunity with local population abundances $n_1$ and $n_2$. We define the local RNs as $\mathcal {R}_1 = 0.5$, $\mathcal {R}_2 = 1.5$. The mobility fluxes between the two nodes are described by the connection matrix

$$\begin{aligned} \textbf{C} = \begin{pmatrix} 1-\xi _1&  \xi _2 \\ \xi _1&  1-\xi _2 \end{pmatrix} , \end{aligned}$$

where $\xi _1$ and $\xi _2$ quantify the outgoing mobility from either of the two nodes.

We first tested the influence of the connectivity of the system on the value of the global effective RN, $\mathcal {R}^G(t)$. Specifically, we investigated how $\mathcal {R}^G(t)$ relates to the largest local effective RN and to the weighted mean of the local effective RNs (where the weights are assumed equal to the proportion of the total resident population in the corresponding node). Our results suggest that

14 $$\begin{aligned} \dfrac{\sum _j \mathcal {R}_j(t) n_j}{\sum _j n_j} \le \mathcal {R}^G(t) \le \max _j \mathcal {R}_j(t), \end{aligned}$$

where $n_j$ is the population of the *j*-th community, and $\mathcal {R}_j(t)$ is its local effective RN.

**Fig. 4:**
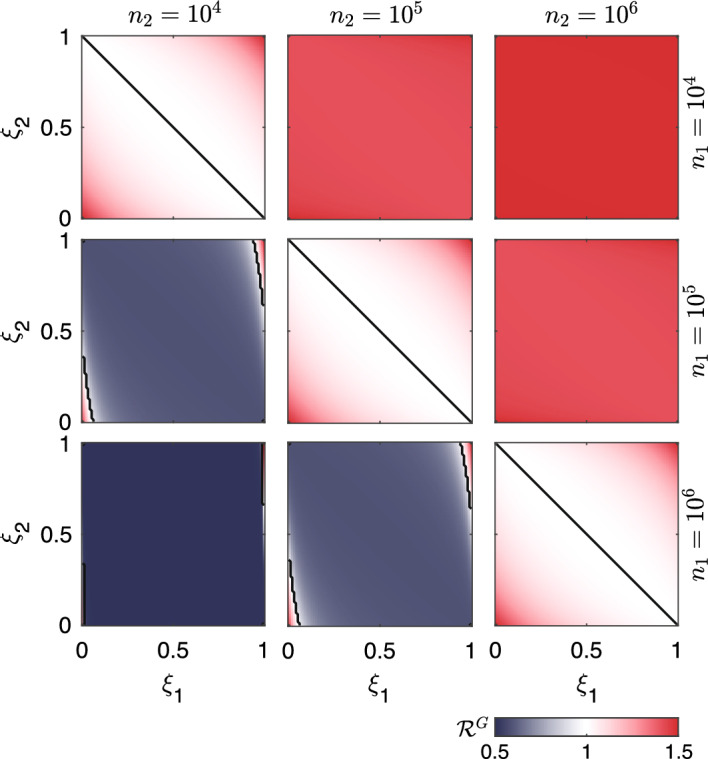
Global RN in a simplified network of two nodes as a function of the proportion of mobile individuals. The black lines correspond to the unit threshold of the global RN. Different population size combinations are tested in the different panels. The two nodes have local RNs $\mathcal {R}_1=0.5$ and $\mathcal {R}_2=1.5$. Mobility fluxes are defined through the coefficients of outgoing mobility from each node ($\xi _1$ and $\xi _2$). Figure after Trevisin et al. [60] (color figure online)

The first result we report (Fig. 4) is that the global RN may take sub-unit values even if one node has a local RN above the unit threshold. Under such scenario, the number of expected infections does not grow indefinitely over time, but rather tends to zero asymptotically. The global RN is indeed defined as a function of not only the local RNs, but also the local populations and the mobility fluxes among the interconnected communities. We find that the global effective RN strongly depends on the differences in population size between the two nodes and the configuration of the outgoing fluxes. Indeed, for all the tested parameter combinations, the global RN appears to be maximum (i.e., equal to 1.5 in our numerical example) for either zero or full outgoing mobility (i.e., when the new infections in each node depend only on the active cases in another, unique node). For well-mixed mobility fluxes (both $\xi$ equal to 0.5, where $\xi$ represents the proportion of outgoing mobility from each node), the global effective RN takes its minimum value, which is equal to the population-weighted average of the local RNs. We find singularities when only one of the $\xi$ is null. Because of the definition of the spatial exposure matrix $\textbf{Z}$, in such cases the active population in either of the two nodes is null, which prevents the computation of the epidemiological indices. Finally, a central symmetry holds for all combinations of the two mobility-related parameters. When different configurations of the local population sizes are tested, the lower bound of the global RN changes following the relationship shown in Eq. (14). A larger population in the node with the largest local RN results in higher values of the global RN across the tested mobility combinations, whereas the opposite induces a drastically lower minimum value of the global RN.

**Fig. 5:**
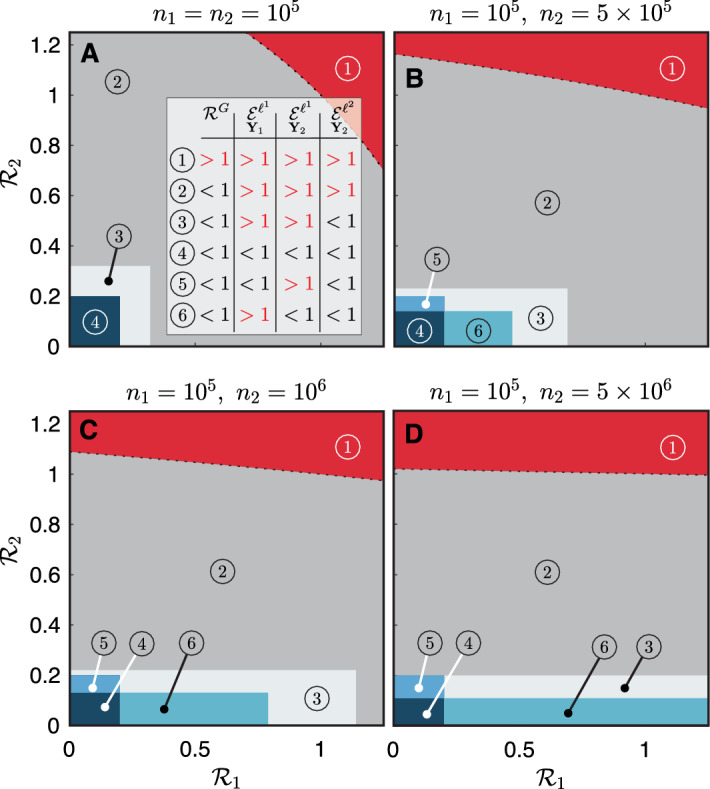
Comparative analysis of the epidemiological indices in a metacommunity with two nodes. Dependence on the two local RNs of the global RN, the $\ell ^1$-norm of both observed variables ($\textbf{Y}_1$ and $\textbf{Y}_2$, respectively the total number of infectious individuals and the prevalence of infectious individuals in each local population), and the $\ell ^2$-norm epidemicity index (based on variable $\textbf{Y}_2$). Colors code different configurations of the epidemiological indices, leading to different short- and long-term disease transmission scenarios (inset of panel **A**). Specifically, red denotes endemic conditions (1), dark gray represents epidemic but non-endemic conditions (2), light gray represents a scenario where the system is reactive in the $\ell ^1$-norm applied to both output transformations but not in the $\ell ^2$-norm applied to the $\mathbf {Y_2}$ transformation (3), and the blue hues represent non-reactivity in at least one of the two considered $\ell ^1$-norms (4–6). For both nodes, $\xi _1=\xi _2=0.25$. Figure after Trevisin et al. [60] (color figure online)

Moreover, we tested the impact of the two local RNs with fixed mobility fluxes ($\xi _1=\xi _2=0.25$) for different configurations of the population distribution (Fig. 5). We found that both local RNs and population sizes substantially impact the values of the considered epidemiological indices (highlighting whether the system may asymptotically converge to a stable DFE and, if so, whether this equilibrium is reactive). In a simple scenario with equal populations in the two nodes, the condition for the asymptotic stability of the DFE ($\mathcal {R}^G<1$, which applies when both local RNs are $<1$ or when one is above the unit threshold and the other is sufficiently below it) does not automatically guarantee non-reactive conditions. Indeed, panel A of Fig. 5 shows that additional thresholds for both local RNs must be met to guarantee that both the $\ell ^1$- and the $\ell ^2$-norm epidemicity indices take sub-threshold values, similarly to previous finding for continuous-time systems [18]. When the population is heterogeneously distributed (panels B–D), with one local population being substantially larger than the other, the global RN almost uniquely depends on the RN of the most populated node, with little dependence on the other local RNs. The global RN displays high sensitivity to the largest local RN whenever the $n_2/n_1$ ratio increases, while this behaviour is less pronounced for the considered epidemicity indices. However, when the population sizes are extremely different from each other (e.g., in the case of Fig. 5D, where $n_2/n_1=50$), then the epidemicity indices computed on the prevalence of infectious individuals acquire an almost exclusive dependence on the RN corresponding to the largest local population. Finally, the threshold for the $\ell ^1$-norm epidemicity never changes and is slightly larger than 0.2, which is consistent with previous spatially-implicit investigations [63].

We also stress that, for given combinations of the two local RNs, there exists a scenario where the $\ell ^1$-norm epidemicity index is below the reactive threshold and its $\ell ^2$ analogue is not. Under our output transformation matrices $\textbf{W}_1$ and $\textbf{W}_2$, this implies that the total number of infectious people is increasing, yet the $\ell ^2$-norm of the local prevalences of infectious individuals is decreasing. Another interesting result concerns the comparison between the $\ell ^1$-norm computed on the total number of infectious individuals (see Fig. 5, where non-reactivity is represented by zones 4 and 5) and that related to their prevalence in each local population (zones 4 and 6 in Fig. 5). The two observed variables do not share their reactivity thresholds unless all population sizes are equal. Indeed, whenever one node has a population that is substantially larger than the other node’s, the threshold of the RN above which reactive conditions on the prevalence of infectious individuals apply decreases substantially. In our two-node example, it can be seen that this threshold is halved when the most populous node has a fifty-fold larger population than the other, as shown in Fig. 5D. Similarly to the $\ell ^2$-norm, we also remark that the reactivity of the output variable $\textbf{Y}_2$ as measured with the $\ell ^1$-norm is almost uniquely dependent on the RN of the node with the highest value when substantially different population sizes are involved, as shown by the elongated shapes of the regions marked as 6 in Fig. 5.

#### Some results for a generic metacommunity

More generally, we assume that the metacommunity is composed of *N* connected local communities, whose population abundance is randomly generated from a Zipf distribution with an exponent equal to $-2$, a value in line with previous estimates e.g., [66], and a minimum value generated from a uniform distribution with support between $10^4$ and $10^5$ to mirror the typical population size of a mid-sized city. The number of communities is also drawn from a discrete uniform distribution whose support includes all natural numbers in [2, 10]. This choice was made to contain the computational burden arising from a larger number of connected nodes. The proportion of outgoing mobility $\xi _l = \sum _{j\ne l} C_{lj}$ is drawn from a normal distribution with reflecting boundaries (to ensure values between 0 and 1) and prescribed mean/standard deviation values. For mobile individuals, the probabilities of reaching different destinations are randomly generated from a uniform 0–1 distribution and normalized to $\xi _l$. The local effective-RNs are generated from a log-normal distribution with a mean of 1.05 and a standard deviation of 0.33, whose parameters are generated by observing real-world COVID-19 effective reproduction number timeseries in several countries (see [67]).

**Fig. 6:**
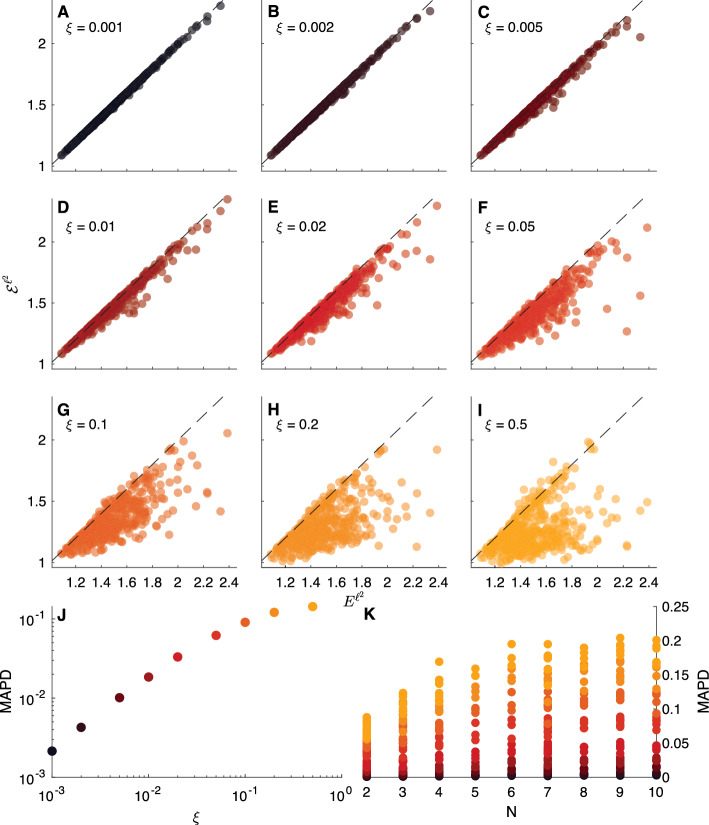
Impact of increasing outgoing mobility fluxes on the $\ell ^2$-norm discrete epidemicity index based on the prevalence of infectious people. **A**-**I**: Scatter plots of the disconnected $\ell ^2$-norm epidemicity index $E^{\ell ^2}$ versus the spatially connected epidemicity index $\mathcal {E}^{\ell ^2}$ for different values of the outgoing mobility parameter $\xi$. The dashed black lines represent the identity relationship. **J**: MAPD between the values of the connected and disconnected $\ell ^2$-norm epidemicity index, as a function of outgoing mobility $\xi$. **K**: As in the left-bottom panel, but MAPD is shown as a function of both outgoing mobility $\xi$ (color coded) and the number of interconnected communities *N*. Figure after Trevisin et al. [60] (color figure online)

We measured how the $\ell ^2$-norm epidemicity index changes when higher mobility fluxes (as determined by the coefficients of outgoing mobility $\xi _j \ (j=1,\dots ,N)$) depart from a baseline configuration without mobility. We found (Fig. 6, panels A–I) lower values of the $\ell ^2$-norm epidemicity index for increasing values of the outgoing mobility parameter $\xi$, which is also reflected in the growth of the mean absolute percentage deviation (MAPD, Fig. 6J) with respect to the scenario without mobility. We also note that, for larger outgoing mobility fluxes ($\xi >0.05$), an increase in the number of the interconnected communities further amplifies the deviation from the no-mobility scenario (Fig. 6K).

## Applications to real cases: cholera in Haiti and COVID-19 in Italy

In this section, we will show how the above theory can be fruitfully applied to some real epidemics. As for water-related diseases we will use cholera as the focus disease, while for air-borne diseases we will consider COVID-19. In both cases we will make reference to specific geographical networks: hydrology and mobility for cholera in Haiti [47] and mobility for COVID-19 in Italy [18, 38, 68].

### The 2010 cholera epidemic in Haiti

Cholera is an infectious waterborne disease caused by contamination with *Vibrio cholerae*, a bacterium that colonizes the intestine after ingestion and can cause, if untreated, severe dehydration and electrolyte loss resulting at times in the death of the host. As infected individuals shed pathogens through their faeces, high bacterial loads may reach communities’ water supplies and fuel the infection cycle in settings where basic sanitation is lacking.

**Fig. 7:**
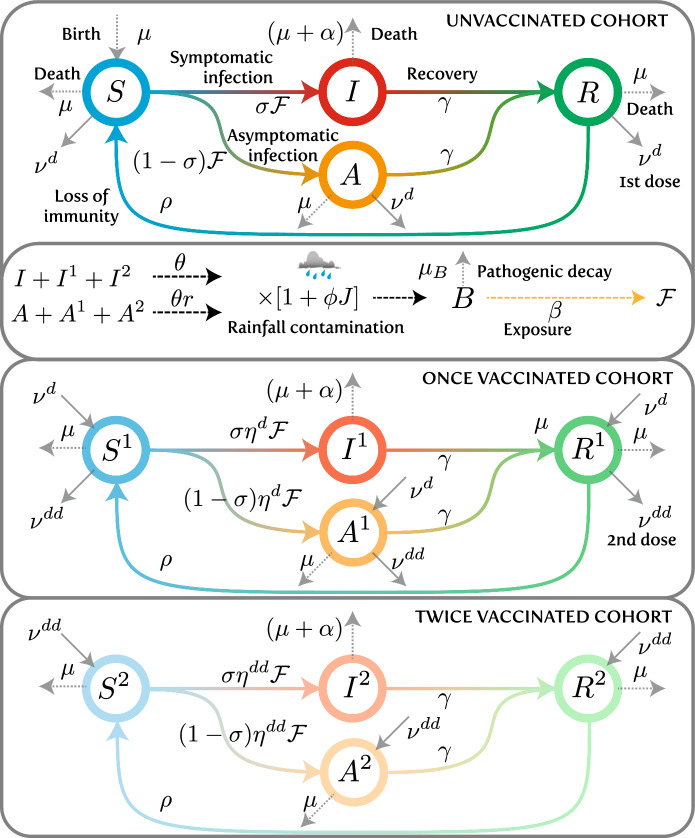
Schematic representation of the model used to analyze the 2010s Haitian cholera outbreak. Parameter $\rho$ here refers to the loss of immunity rate and not the spectral radius operator. Figure after Trevisin et al. [19]

One of the most severe cholera outbreaks in recent history struck Haiti just months after a catastrophic earthquake had hit the country on January 12, 2010, imposing a major death toll and effectively destroying whatever sanitation and transportation infrastructures pre-existed. Sadly, cholera—at that time unknown to the island for more than two centuries—was accidentally seeded into the country from Nepal, where a severe cholera outbreak had been ongoing [69, 70]. Given the lack of basic sanitation infrastructure and the obvious unpreparedness of the population, cholera quickly propagated through the whole country showing remarkable spatial signature [28, 47, 69–75]. In 2013, the Haitian health authorities, jointly with public and private partners, teamed up to establish a protocol for eradicating the disease from Haiti [70]. The chosen strategy consisted of targeted water, sanitation and hygiene (hereafter WaSH) interventions, deployed intensively and in a capillary manner [70], combined with a vaccination campaign of large proportions—and difficult logistics [74, 76]. WaSH interventions were carried out by rapid response teams depending on an alert system and involved, e.g., educational sessions for the local population, distribution of chlorination and sanitation products, nursing support, and additional prophylaxis. Here, we move from a detailed understanding of the effects of each WaSH intervention carried out in space and time in Haiti, to investigate whether reliable prognostic indices exist. To this end, we shall adapt an extensively used family of spatio-temporal epidemiological models [23, 28, 30, 45, 47, 50, 51, 77–79]. With respect to the generic model introduced above to describe the initial phase of waterborne diseases, we considered additional compartments that are necessary to assess the efficacy of the different interventions. The structure of the model is illustrated in Fig. 7.

#### Stability of disease-free equilibrium and next-generation matrix

Our derivation follows the approach traced by a number of relevant contributions [5, 11, 15, 31, 46, 80] and where the epidemiological model accounts for the presence of asymptomatic infectious individuals as well as two cohorts of vaccinated individuals and a compartment of recovered people (possibly losing their immunity). Specifically, we evaluate our key epidemiological indices, i.e., the effective RN and the epidemicity index, in a temporal and spatially explicit manner. The epidemicity index requires the specification of which states-at-infection are included to calculate the norm of the system output *y*. We are addressing the symptomatically infectious compartment with the output being defined as [1, 12]

$$\begin{aligned} {\textbf{y}}(t) = \left\{ I_i(t); \text {, } i =1,..,10 \right\} \end{aligned}$$

whose norm is

$$\begin{aligned} || {\textbf{y}}(t) || = ||{\textbf{I}}(t)||_2. \end{aligned}$$

To further simplify the analysis of the model, shown in Fig. 7, we assume bacteria to hold faster dynamics than the other state variables [81, 82]. As such, we impose the local equilibrium condition

$$\begin{aligned} \dfrac{\partial B_i}{\partial t} =0 , \end{aligned}$$

which allows one to directly compute the bacterial concentration as a linear function of the infectious pool, i.e.,

$$\begin{aligned} B_i(t) = \dfrac{\theta _i(t) I_i(t)}{\mu _B H_i}. \end{aligned}$$

The force of infection can be rearranged accordingly as

15 $$\begin{aligned} \mathcal {F}_i(t) = (1-m) \beta _i(t) \dfrac{\theta _i(t) I_i(t)}{\theta _i(t) I_i(t)+\mu _B H_i} + m \displaystyle \sum _{j \ne i} Q_{ij} \beta _j (t) \dfrac{\theta _j(t)I_j(t)}{\theta _j(t) I_j(t)+\mu _B H_j}. \end{aligned}$$

The derivative of the force of infection with respect to each of the symptomatically infected stages $X^I_k$ ($I_k$) yields

$$\begin{aligned} \dfrac{\partial \mathcal {F}_i(t)}{\partial X^I_k} = {\left\{ \begin{array}{ll} (1-m) \beta _i(t) \dfrac{\theta _i(t) \mu _B H_i}{\left[ \theta _i(t) \mathcal {I}^P_i(t)+\mu _B H_i\right] ^2} &  \text {if } k=i \\ m Q_{ik} \beta _k (t) \dfrac{\theta _k(t)\mu _B H_k}{\left[ \theta _k(t) \mathcal {I}^P_k(t)+\mu _B H_k\right] ^2} &  \text {otherwise} \end{array}\right. } . \end{aligned}$$

One can thus compute the generalized Jacobian matrix that is associated to the modified ODE system along its trajectory. Let $f_p$ be the generic *p*-th ODE equation and $X_q$ the generic *q*-th state variable. The Jacobian matrix ${\textbf{J}}$ is defined as

$$\begin{aligned} j_{pq} = \dfrac{\partial f_p}{\partial X_q} \end{aligned}$$

and ${\textbf{J}} = \left[ j_{pq}\right]$. To compute the effective RN and the epidemicity index, one should only consider the infectious subsystem, which corresponds to the state variables pertaining to the infected stages of the model. The infectious subsystem ${\textbf{y}}$ can be obtained from the algebraic transformation ${\textbf{y}} = {\textbf{W}} {\textbf{x}}$ of the full system state ${\textbf{x}} = \left\{ S_i, I_i, R_i \text {, } i = 1,\dots ,10 \right\}$, with

$$\begin{aligned} {\textbf{W}} = \begin{pmatrix} {\mathbf {0_n}}&{\mathbf {U_n}}&{\mathbf {0_n}} \end{pmatrix} . \end{aligned}$$

The dynamics of the infectious subsystem are thus described by the reduced-order Jacobian

16 $$\begin{aligned} {\mathbf {J^\star }}_t = {\textbf{W}} {\textbf{J}}_t {\textbf{W}}^T = \sigma {\mathbf {F'}}_t{\textbf{S}}- \Phi {\mathbf {U_n}} , \end{aligned}$$

where *T* indicates matrix transposition; ${\mathbf {F'}}$ is the matrix containing the derivatives of the force of infection as defined in Eq. (15); ${\textbf{S}}$, is a diagonal matrix whose nonzero elements represent the abundance of susceptibles individuals in the ten Haitian departments; ${\mathbf {U_n}}$ is the identity matrix of size $n=10$; and $\Phi = \gamma +\alpha +\mu$.

#### Computation of the disease reproduction number

The application of the NGM method to compute the RN [15] in this model starts by rewriting the Jacobian matrix of the system as the sum of the transmission matrix ${\textbf{T}}_t$ and the transition matrix ${\mathbf {\Sigma }}_t$

$$\begin{aligned} {\mathbf {J^\star }}_t = {\textbf{T}}_t + {\mathbf {\Sigma }}_t. \end{aligned}$$

The transmission matrix includes the term related to the rate of appearance of new infections and is computed as

$$\begin{aligned} {\textbf{T}}_t = \sigma {\mathbf {F'}}{\textbf{S}} , \end{aligned}$$

while the transition matrix contains the other infectious-related terms, i.e.,

$$\begin{aligned} {\mathbf {\Sigma }}_t = - \Phi {\mathbf {U_n}}. \end{aligned}$$

One can thus define the NGM as

$$\begin{aligned} {\textbf{K}}_t = -{\textbf{T}}_t \left( {\mathbf {\Sigma }}_t \right) ^{-1} \end{aligned}$$

and finally obtain the effective RN as the spectral radius of the NGM, i.e.,

$$\begin{aligned} \mathcal {R}_t(t) = \rho \left( {\textbf{K}}_t\right) . \end{aligned}$$

#### Computation of the epidemicity index

A reactive transient behavior is connected to the sign of the derivative of the norm of the system output (i.e., in our case, the infectious subsystem) being positive, that is,

$$\begin{aligned} \left. \dfrac{d||{\textbf{y}}||}{dt} \right| _{t=0}>0 . \end{aligned}$$

If we use the $\ell _2$ norm to measure the amplitude of the perturbation, the above condition is verified when the largest eigenvalue of the Hermitian part of the reduced-order Jacobian (Eq. (16)) is positive, namely,

$$\begin{aligned} \lambda _{\text {max}} \left( {\textbf{H}}({\mathbf {J^\star }})_t\right) >0 , \end{aligned}$$

where the Hermitian matrix is defined as ${\textbf{H}}({\mathbf {J^\star }}_t) = ( {\mathbf {J^\star }}_t+{\mathbf {J^\star }}_t^T)/2$. We compute the epidemicity index as the largest eigenvalue of the aforesaid Hermitian matrix, i.e.,

$$\begin{aligned} e_t(t) = \lambda _{\text {max}} \left( {\textbf{H}}({\mathbf {J^\star }}_t)\right) . \end{aligned}$$

#### Derivation of the analytical relationship between the reproduction number and the epidemicity index

The RN and the epidemicity index can be mathematically related through some algebraic manipulations. Let us now consider a model with a single unvaccinated cohort, out of simplicity. The infectious subsystem of the Jacobian matrix would read

$$\begin{aligned} {\textbf{J}}_t^\star = \sigma {\mathbf {F'}} {\textbf{S}} - \Phi {\mathbf {U_n}} , \end{aligned}$$

where ${\mathbf {F'}}$ is defined in Eq. (5.1). The transmission matrix is defined as

$$\begin{aligned} {\textbf{T}}_t^\star = \sigma {\mathbf {F'}} {\textbf{S}} , \end{aligned}$$

while the transition matrix is simply defined as

$$\begin{aligned} {\mathbf {\Sigma }}_t = - \Phi {\mathbf {U_n}}. \end{aligned}$$

The assumption that the infectious pool is much smaller compared to the whole population of a given node *i* ($I_i \ll H_i$) allows us to simplify Eq. (5.1) as

$$\begin{aligned} \dfrac{\partial \mathcal {F}_i(t)}{\partial X^I_k} = {\left\{ \begin{array}{ll} (1-m) \beta _i(t) \dfrac{\theta _i(t)}{\mu _B H_i} &  \text {if } k=i \\ m Q_{ik} \beta _k (t) \dfrac{\theta _k(t)}{\mu _B H_k} &  \text {otherwise} \end{array}\right. } , \end{aligned}$$

which can be rewritten in matrix form as

$$\begin{aligned} {\mathbf {F'}} = \left[ (1-m) {\mathbf {U_n}} + m {\textbf{Q}} \right] {\mathbf {\beta }}(t) {\mathbf {\theta }}(t) \dfrac{1}{\mu _B} {\textbf{H}}^{-1} , \end{aligned}$$

where ${\mathbf {\beta }}(t)$ and ${\mathbf {\theta }}(t)$ are diagonal matrices whose nonzero terms are the exposure $\beta _i(t)$ and the shedding $\theta _i(t)$ rates for each department *i*. Let’s now proceed with the computation of the basic RN $\mathcal {R}_0$ and the basic epidemicity index $e_0$. In this case, the following properties hold: ${\mathbf {\beta }}(t) = \beta _0 {\mathbf {U_n}}$, ${\mathbf {\theta }}(t) = \theta _0 {\mathbf {U_n}}$, and ${\textbf{S}}\simeq {\textbf{H}}$, a direct consequence of the lack of immunity buildup and control measures in the concerned population at the beginning of an epidemic. As a result, matrix ${\mathbf {F'}}$ simplifies to

$$\begin{aligned} {\mathbf {F'}} = \dfrac{\beta _0 \theta _0}{\mu _B} \left[ (1-m) {\mathbf {U_n}} + m {\textbf{Q}} \right] {\textbf{H}}^{-1} , \end{aligned}$$

while matrix ${\mathbf {T_0}}$ simplifies to

$$\begin{aligned} {\mathbf {T_0}} = \omega \left[ (1-m) {\mathbf {U_n}} + m {\textbf{Q}} \right] , \end{aligned}$$

where $$\omega = \sigma \beta _0 \theta _0 / \mu _B$$. Letting

$$\begin{aligned} {\textbf{M}} = \left[ (1-m) {\mathbf {U_n}} + m {\textbf{Q}} \right] , \end{aligned}$$

we obtain ${\mathbf {J^\star _0}}=\omega {\textbf{M}}-\Phi {\mathbf {U_n}}$ and ${\mathbf {T_0}}=\omega {\textbf{M}}$. The basic RN is thus given by

$$\begin{aligned} \mathcal {R}_0 = \rho ({\mathbf {K_0}}) = \rho (-{\mathbf {T_0}}({\mathbf {\Sigma _0}})^{-1}) = \dfrac{1}{\Phi }\rho ({\mathbf {T_0}}) = \dfrac{\omega }{\Phi } \rho ({\textbf{M}}) = \dfrac{\omega }{\Phi } \end{aligned}$$

because matrix ${\textbf{M}}$ is row-stochastic, hence its spectral radius is equal to one. As for the basic epidemicity index, we have

$$\begin{aligned} e_0 = \rho (\textrm{H}(\omega {\textbf{M}}-\Phi {\mathbf {U_n}})) = \omega P\left( \dfrac{{\textbf{M}}+{\textbf{M}}^T}{2}\right) - \Phi \end{aligned}$$

because of the property

$$\begin{aligned} \left| \textrm{H}({\mathbf {J_0^\star }} - \lambda {\mathbf {U_n}})\right| = \left| \omega \dfrac{{\textbf{M}}+{\textbf{M}}^T}{2} - \Phi {\mathbf {U_n}} - \lambda {\mathbf {U_n}} \right| = \left| \omega \dfrac{{\textbf{M}}+{\textbf{M}}^T}{2} - \mu {\mathbf {U_n}} \right| , \end{aligned}$$

where $\mu = \lambda + \Phi$. By setting $\bar{\mu } = P \left( \dfrac{{\textbf{M}}+{\textbf{M}}^T}{2} \right)$, we obtain

$$\begin{aligned} e_0 = \Phi \bar{\mu } \mathcal {R}_0- \Phi . \end{aligned}$$

The general case where we consider effective RNs and epidemicity indices cannot be solved analytically because matrix ${\textbf{T}}_t$ contains parameters ${\mathbf {\beta }}$, ${\mathbf {\theta }}$, and the state variable ${\textbf{S}}$ that are spatially and temporally heterogeneous. Let $\tau _t = \rho ({\textbf{T}}_t) = P\left( \sigma {\textbf{M}} {\mathbf {\beta }}(t) {\mathbf {\theta }}(t) \dfrac{1}{\mu _B} {\textbf{H}}^{-1} {\textbf{S}}\right)$ and $\eta _t = \rho (\textrm{H}({\textbf{T}}_t))$. Note that the relation

$$\begin{aligned} e_t = \Phi \dfrac{\eta _t }{\tau _t}\mathcal {R}_t- \Phi \end{aligned}$$

still holds. This equation allows us to determine the threshold value that the RN should take for the system to attain a stable, non-reactive disease-free equilibrium ($e_t \le 0$), namely

$$\begin{aligned} \mathcal {R}_t \le \dfrac{\tau _t }{\eta _t}. \end{aligned}$$

In the example of the Haitian cholera epidemic, the mean across all times of the ratio $\tau _t/\eta _t$ is 0.729.

#### Mathematical analysis of the Haitian epidemic

**Fig. 8:**
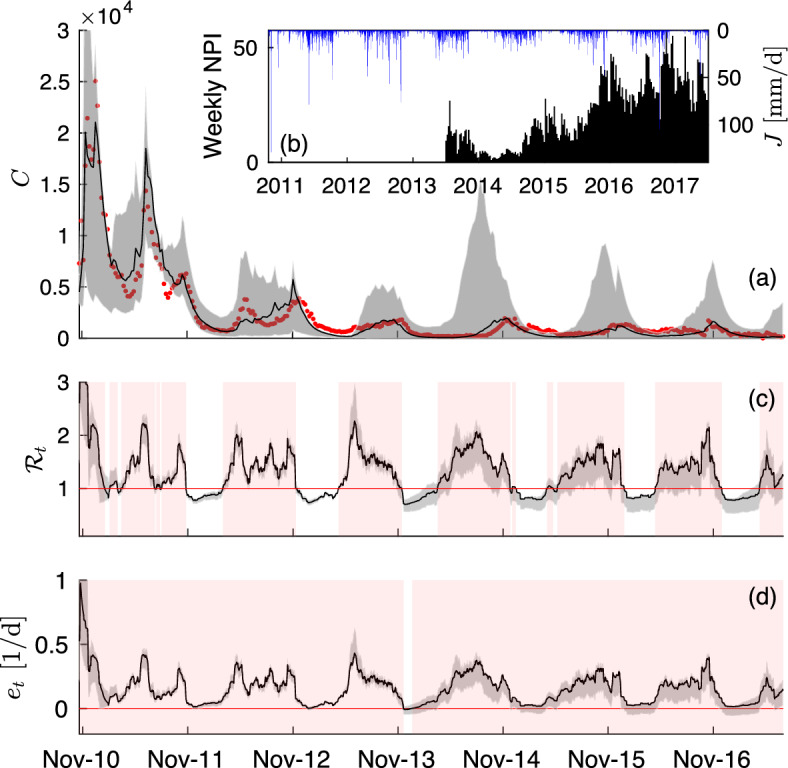
Temporal dynamics of cholera incidence, effective RN, and epidemicity index in Haiti. **a** Number of recorded (red dots) and simulated (black solid line) weekly infections in Haiti between October 2010 and June 2017. **b** Weekly Non-Pharmaceutical Interventions (NPIs) deployed (gray bars) and incoming rainfall averaged over the whole Haitian territory (blue bars). **c** Effective RN, smoothed over a four-weeks span. **(d)** Epidemicity index, also smoothed over a four-weeks span. Gray shadings in panels (**a**), (**c**), and (**d**) denote the 50% confidence interval obtained by sampling several times (SI) the posterior distribution of the model parameters. In (**c**) and (**d**), a red background indicates super-threshold values of either the effective RN ($\mathcal {R}_t > 1$) or the epidemicity index ($e_t > 0$). Figure after Trevisin et al. [19] (color figure online)

**Fig. 9:**
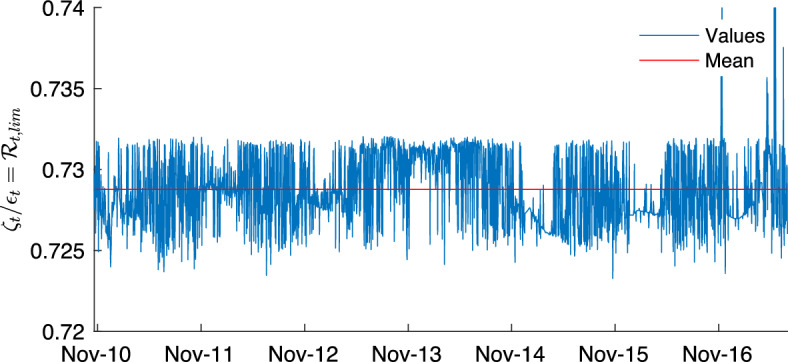
Timeseries of the temporal variation of the ratio between the spectral radius of the transmission matrix $\tau _t$ and that of its Hermitian part $\eta _t$ for the Haiti cholera epidemic. The mean of the timeseries is shown in red. This ratio represents the upper bound of the effective RN that would guarantee the effective epidemicity index to become negative. Figure after Trevisin et al. [19] (color figure online)

Figure 8 shows the computed timeseries of the two epidemiological indices introduced above and computed using a calibrated more complex model [19], the effective RN $\mathcal {R}_t$ (Sect. 5.1) and the epidemicity index $e_t$ (Sect. 5.1). The effective RN exhibits frequent upcrossing of the unit threshold due to seasonal infection revamping via increased exposure to the disease brought in by washout of pathogens by tropical rainfall patterns. The epidemicity index $e_t$ displays fluctuations synchronized to those exhibited by $\mathcal {R}_t$, yet, critically, almost always inside a range of positive values. This indicates that dry seasons, albeit characterised by a sub-threshold $\mathcal {R}_t$, still retain epidemic potential embedded in positive epidemicities. This in turn implies that a perturbation of the system by new infections may result in a possibly significant transient response by coalescence of sub-threshold local outbreaks. A linear regression between the epidemicity indices and the corresponding effective RNs reveals almost perfect correlation ($R^2 \approx 1$) between $e_t(t)$ and $\mathcal {R}_t(t)$, i.e.,

$$\begin{aligned} e_t(t) = \beta _0 + \beta _1 \mathcal {R}_t(t) , \end{aligned}$$

with coefficients $\beta _0 = -0.204 \pm 0.01$ [d$^{-1}$] and $\beta _1=0.280 \pm 0.01$ [d$^{-1}]$. It is interesting to note that, according to the attempted linear regression, $\beta _0 = - \Phi = - (\mu +\alpha +\gamma )$ and $\beta _1 = \Phi \overline{\eta _t/\tau _t}$, the latter term corresponding to the average value of the ratio between the spectral radius of the Hermitian part of the transmission matrix and that of the transmission matrix itself. Only small oscillations of the ratio around the mean can be observed, which explains the very high correlation between $R_t$ and $e_t$. The variation of the ratio is influenced by the evolution of the real-time epidemic and the changing values of parameters. Figure 9 displays the inverse of the ratio, which represents the subthreshold value of the RN that would guarantee a negative epidemicity index. This condition has been met only in a tiny temporal window (January 2014).

### The 2020 COVID-19 epidemic in Italy

In what follows, we will resort to the COVID-19 pandemic in Italy between January 24, 2020 and October 15, 2021 as a testbed for our analytical framework. The rationale behind this choice, in particular concerning the selected time window, is that suspected cases linked to the BA.1 (Omicron) variant, which substantially altered the epidemiological parameters (such as the distribution of generation times), were detected in Italy shortly after this period. Extending the temporal window of analysis would thus require a multi-variant approach [83], which lies outside the scope of this paper. We consider $N=20$ local communities, corresponding to the regions of Italy (first-level administrative divisions). Epidemiological data is retrieved from the Italian Department of Public Protection (https://github.com/pcm-dpc/COVID-19). It should be noted that, for the sake of standardization, data from the autonomous provinces of Trento and Bolzano are merged in a single region Trentino-Sudtirol (while they correspond to different NUTS-2 entities for statistical purposes). To evaluate matrices $\textbf{C}$ and $\textbf{Z}$, as well as the local effective RNs, we resort to the same technique implemented in [59], which is based on the first equation of system (11), with $I_{j,1}$ representing the new infections observed in node *j* at time *t*. Note that the RNs calculated in this way do not correspond to the global RN introduced above; rather, they represent sequences of regional-level RNs that also account for the local connectivity patterns of each region. They also implicitly account for the decrease of susceptible prevalence due to the implementation of the first vaccination which started at the beginning of 2021. The pre-pandemic connectivity matrix is estimated through mobility data obtained from the Italian Institute of Statistics (ISTAT) (accessible at https://www.istat.it/it/archivio/139381) and updated during the COVID-19 pandemic through the "Workplace mobility" information provided by the Google Community Mobility Reports (https://www.google.com/covid19/mobility/). The computation of the aforementioned matrices is identical as in [59], except for a coarser spatial resolution (regional vs. provincial) being chosen to compensate a wider spatial domain (national vs. regional).

**Fig. 10:**
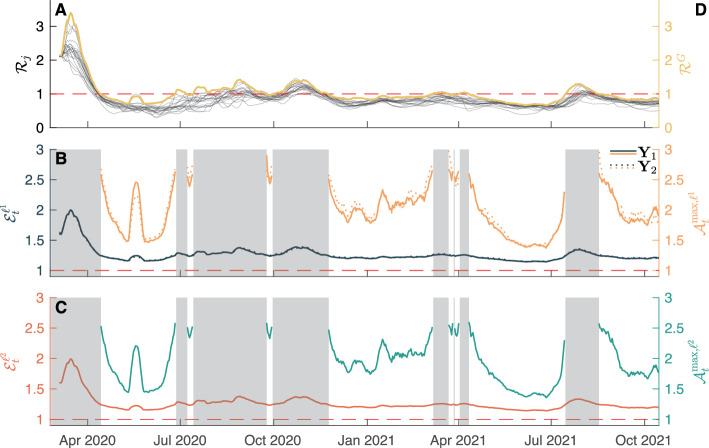
Reactivity analysis of the COVID-19 epidemics in Italy. **A**: Local effective RNs inferred from each region of Italy (thin gray lines) and global effective RN (thick yellow line). **B**: $\ell ^1$-norm epidemicity index (left axis, dark blue) and maximum value of the amplification envelope (right axis, yellow) of the variables $\textbf{Y}_1$ (total number of infectious individuals in each community) and $\textbf{ Y}_2$ (prevalence of infectious individuals in each community). **C**: $\ell ^2$-norm epidemicity index (left axis, red) and maximum value of the amplification envelope (right axis, green). In panels **B** and **C**, gray patches denote $\mathcal {R}^G(t)>1$ (in which case the amplification envelope grows unbounded over time). The Bayesian method used to compute the local effective RNs produces numerically close values of the local effective RNs at the beginning of the COVID-19 epidemic. This reflects the fact that the epidemic wave does not affect all nodes at the same time. Figure after Trevisin et al. [59] (color figure online)

In the following, we provide a practical example of the computation of the epidemicity indices in a realistic spatially explicit setting. Our results are summarized in Fig. 10. We observe that the global effective RN is quite close to the largest local effective RN for most of the considered period (panel A), owing to the fairly low mobility coefficients (the largest value reached by any $\xi _j$ is equal to 0.045). The global effective RN, depending on whether the epidemic is waning or revamping, oscillates across the unit threshold, which leads to the maximum value of the amplification envelope (panels B and C) taking non-finite values at times. In contrast, the epidemicity indices always take larger-than-unit values, regardless of the chosen norm. Given our choice of transformation matrices $\textbf{W}_1$ and $\textbf{W}_2$, we find that the epidemicity indices computed in the two considered algebraic norms are very close ($0.4\%$ mean absolute percentage deviation), with the values computed with the $\ell ^1$-norm being slightly larger than their $\ell ^2$-norm counterparts. An analysis of the epidemic subset shows that, for both norms and output transformations, all regions were reactive during the whole timespan of our analysis. This finding suggests that suitable perturbations to the DFE (e.g., in the form of imports of cases with an age of infection close to the peak in transmissibility) could have produced transient (if $\mathcal {R}^G(t) < 1$) or sustained (if $\mathcal {R}^G(t) > 1$) epidemic outbreaks regardless of the region(s) where the perturbation was localized and the time when it occurred. We also compared (Fig. 10B) the $\ell ^1$-norm epidemicity indices based on the total number of infectious individuals and their prevalence within the local populations. We found that, although some differences can be spotted, they are not quantitatively large, with a mean absolute percentage deviation (with respect to $\textbf{Y}_1$) of $0.56\%$ for the $\ell ^1$-norm epidemicity index and of $5.56\%$ for the maximum value of the corresponding amplification envelope.

**Fig. 11:**
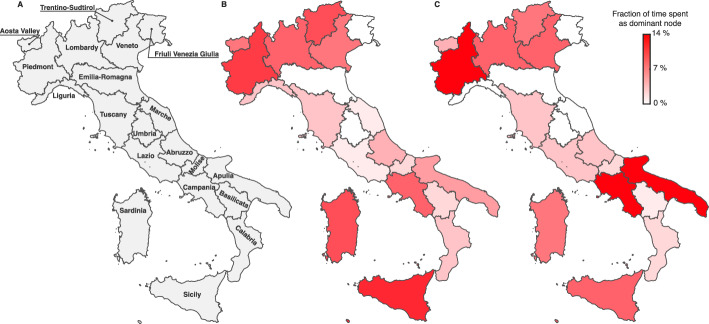
Share of the time each Italian region spends as dominant node from an epidemicity perspective during the the COVID-19 epidemic. **A** Map of Italy and its regions. Please note that, for graphical purposes, the position of Sardinia has been slightly displaced eastwards and some Mediterranean islands have been removed. **B** Fraction of time spent as dominant node in the epidemic subset computed with the total number of infectious individuals in each region (observed variable $\textbf{Y}_1(t)$ with the $\ell ^1$-norm). **C** Fraction of time spent as dominant node computed on the prevalence of infectious individuals in each region (observed variable $\textbf{Y}_2(t)$ with the $\ell ^1$-norm). Figure after Trevisin et al. [59] (color figure online)

A correlative analysis between each local effective RN, the global effective RN, and the different epidemicity indices shows that each local effective RN correlates in a similar way to each of the other considered epidemiological indices. We find that both the global effective RN and the largest local effective RN correlate almost perfectly with the $\ell ^1$-norm epidemicity computed on output variable $\textbf{Y}_1$, with a correlation coefficient of 0.9994 and 1, respectively. They also correlate with the $\ell ^2$-norm epidemicity calculated on the prevalence of infectious individuals in the local populations ($\textbf{Y}_2$), with coefficients of 1 and 0.9994, respectively. Because the COVID-19 epidemic in Italy has often produced synchronous effects in many Italian regions, there also is high correlation between each local effective RN and both the global effective RN and the $\ell ^2$-norm epidemicity index, with coefficients ranging between 0.84 (Molise) and 0.98 (Lazio). However, it can be seen that the fraction of time each region dominates from an epidemicity perspective (e.g., a perturbation in that node of the metacommunity would produce the largest short-term growth in the system output) is different for the two compared variables, owing to variations of the $\eta _j(t)$ coefficient (Fig. 11). These differences in the share of time each node dominates across the two different output variables also reflect different epidemicity thresholds for the local RN to guarantee non-reactive conditions. While these variations in the threshold values are not particularly strong, especially given the homogeneity in mobility fluxes across the different Italian regions, they do exist. The region that requires the lowest RN threshold to warrant non-reactive conditions is Apulia (with a limit of 0.202) which is also the region with the highest share of time as the dominant node (Fig. 11). The region that would require the least stringent threshold is Molise (whose limit for the RN is 0.226).

**Fig. 12:**
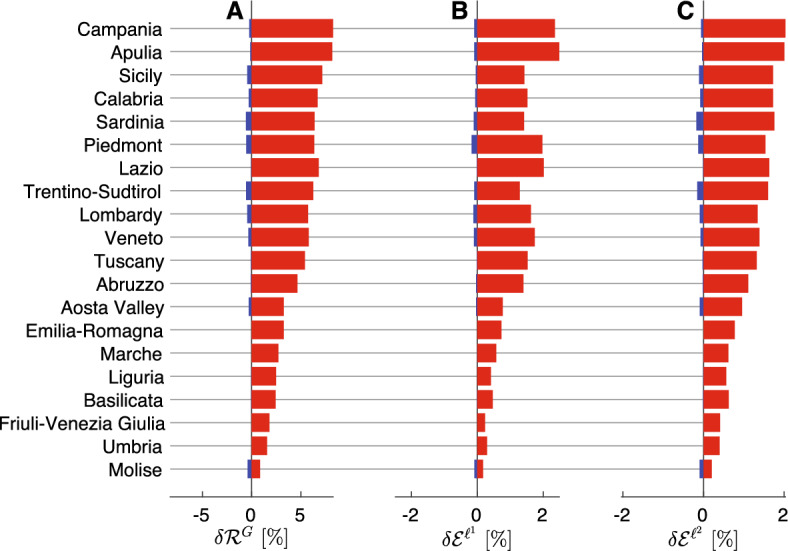
Sensitivity analysis of selected epidemiological indicators evaluated in the context of the COVID-19 pandemic in Italy. Mean percentage deviation of the time series of the global RN (panel **A**), and of the $\ell ^1$- and $\ell ^2$-norm epidemicity indices (panels **B** and C, respectively) of the prevalence of infected individuals (observed variable $\textbf{Y}_2$), using a $\pm 20\%$ variation of the RN time series in each region with respect to the baseline estimates shown in Fig. 10. The effects of positive perturbations are shown in red, whereas negative perturbations are shown in blue. Figure after Trevisin et al. [59] (color figure online)

Finally, to disentangle the effect of each region’s local RN on the global RN and the $\ell ^2$-norm epidemicity index, we carried out a sensitivity analysis where we added a $\pm 20 \%$ variation to each time series of local effective RNs (as shown in Fig. 10A), one at a time. This analysis is shown in Fig. 12. Positive $20\%$ variations of one local RN often result in a positive $5-10\%$ variation of the global RN and a positive $0.5-2\%$ variation on the epidemicity indices, with similar effects on each index. We also find a negative correlation (with coefficient $-0.90$) between positive variations of the $\ell ^1$-norm of the epidemicity index based on the prevalence of infectious individuals in each node and the nodal thresholds for the RN guaranteeing non-reactivity. On the other hand, only reducing one local effective RN at a time produces little to no impact on any of the considered epidemiological indices. This is also justified by all regions being always well above the reactivity threshold.

## Conclusions

Today, society demands rapid restarts of socioeconomic activities after outbreaks of infectious disease, and thus effective methodologies to selectively relax all kinds of containment measures, like lockdowns enforced to control the spread of infections. Here, we have reviewed tools that predict the short-term dynamics and long-term fate of emerging infectious diseases, and the effect of their containment measures in space and time. In particular, we have reviewed novel tools that evaluate the potential for transient increases of epidemics in space and time even when eventually doomed to disappearance.

In fact, it becomes particularly important to identify under what conditions transient epidemiological waves can be observed in the system, regardless of whether or not the disease can self-sustain in the long run (i.e., remain endemic). Building on the idea of reactivity originally defined by Neubert and Caswell [1], the concept of g-reactivity introduced by Mari et al. [12] allowed for a better focus on whether the epidemiological system can allow for such transient waves of infection. The epidemicity index $\mathcal {E}$ ([12]) is nothing more than the dominant eigenvalue of the Hermitian part of a disease-relevant matrix, which combines the intrinsic dynamics of the epidemiological system (properly lin444earized) with infection-specific measures that we fear may grow over time even if only transiently, leading to an epidemic wave.

In the course of an epidemic, say *in medias res*, in order to understand whether outbreaks of infection may flare up in space and time, the press (but also researchers) often refer to reproduction numbers (RNs), starting with the basic RN ($\mathcal {R}_0$) derived through the next-generation matrix approach of Diekmann et al. [15]. Although RNs are extremely powerful in determining the ultimate fate of the disease in the (meta)community, they are not appropriate to ultimately tell whether a re-emergence of the disease can occur in the short term. The most intriguing and insidious cases are those in which sub-threshold epidemics might actually occur, i.e., cases where the disease cannot persist in the long run (the disease-free equilibrium is stable, $\mathcal {R}_0<1$), but epidemic outbreaks are possible, i.e., the epidemicity index $\mathcal {E}$ is positive (in continuous-time models) or greater than unity (in discrete-time models).

With reference to waterborne diseases, we reviewed some of our theoretical results using a sufficiently general metacommunity model for waterborne diseases that, when properly adapted to context, has already been successfully used to address real-world cholera epidemics. In it, while human-infecting pathogens are water transported over Optimal Channel Networks (e.g. OCNs), humans are assumed to move through network nodes according to a gravity model. Our combined analysis of epidemicity indices ($\mathcal {E}$) and of reproduction numbers ($\mathcal {R}_0$) revealed that transient epidemic waves can be triggered by appropriate perturbations of the disease free equilibrium even in cases where long-term pathogen establishment is not possible only if human exposure to infective agents or water contamination is sufficiently large. Also, for fixed exposure and contamination conditions, our indices permit to clearly evidence the specific role played by human mobility and by hydrological transport rates of pathogens in determining whether an epidemiological situation is g-reactive or not.

With reference to airborne diseases spreading in metacommunities, we overviewed the formalization in discrete time of an SIR-like epidemiological process of disease transmission in which local communities are characterized by different reproduction numbers and mobility parameters. Discretization in time becomes instrumental here to deal with data that health authorities release at constant temporal intervals (e.g., daily). Although the model presented provides a fairly detailed description of the infection process, as each local population of infected is divided by age of infection, we provide a compact way to analytically calculate the epidemicity index that effectively allows us to understand whether transient epidemics are possible. We also calculated the global reproduction number (RN) following a canonical Next-Generation-Matrix approach. Computing the indices on a synthetic community consisting of only two nodes was sufficient to reveal that the global RN can take sub-unit values even if one node has a local RN above the threshold of unity. Also, it allowed us to delineate how the transient epidemic wave depends on both human mobility between nodes and local population size.

To showcase the importance of our analysis, we applied the methodology to the two important cases of the cholera epidemics in Haiti just after the 2010 earthquake, and to COVID-19 epidemic that hit Italy as the first European country in 2020. Overall, we have provided a coherent framework borne out of recent epidemiological research contributions.

## Data Availability

Not applicable.
